# Alleles of the GRF3-2A Gene in Wheat and Their Agronomic Value

**DOI:** 10.3390/ijms222212376

**Published:** 2021-11-16

**Authors:** Mikhail S. Bazhenov, Anastasiya G. Chernook, Ludmila A. Bespalova, Tatiana I. Gritsay, Nadezhda A. Polevikova, Gennady I. Karlov, Lubov A. Nazarova, Mikhail G. Divashuk

**Affiliations:** 1 Laboratory of Applied Genomics and Crop Breeding, All-Russia Research Institute of Agricultural Biotechnology, 127550 Moscow, Russia; irbis-sibri@yandex.ru (A.G.C.); karlovg@gmail.com (G.I.K.); lpukhova@yandex.ru (L.A.N.); divashuk@gmail.com (M.G.D.); 2 Kurchatov Genomics Center-ARRIAB, All-Russia Research Institute of Agricultural Biotechnology, 127550 Moscow, Russia; 3 National Center of Grain Named after P.P. Lukyanenko, Department of Breeding and Seed Production of Wheat and Triticale, Central Estate of KNIISH, 350012 Krasnodar, Russia; bespalova_l_a@rambler.ru (L.A.B.); t_i_gritsay@mail.ru (T.I.G.); nadyapolevikova@gmail.com (N.A.P.)

**Keywords:** GRF, transcription factor, diversity, NGS, earliness, kernel weight, test weight

## Abstract

The Growth-regulating factors (GRF) are a family of plant-specific transcription factors that have roles in plant growth, development and stress response. In this study the diversity of the *TaGRF3-2A* (TraesCS2A02G435100) gene was investigated in Russian bread wheat germplasm by means of next generation sequencing and molecular markers, and the results compared with those from multiple wheat genome and exome sequencing projects. The results showed that an allele possessing c.495G>T polymorphism found in Bezostaya 1 and designated as *TaGRF3-2Ab*, is connected with earlier heading and better grain filling under conditions of the Krasnodar Krai. *TaGRF3-2Ab* is more frequent among Russian winter wheat cultivars than in other germplasms found in the world, implying that it is adaptive for the Chernozem region. A new rare mutation of the *TaGRF3-2A* was found in the spring wheat cultivar Novosibirskaya 67. The molecular markers developed will facilitate utilization of *TaGRF3-2A* mutations in future agronomic studies and wheat improvement. Albeit *GRF3-2Ab* may be good at maintaining high milling quality of the grain, it should be used with caution in breeding of winter wheat cultivars in the perspective of climate change.

## 1. Introduction

Growth-Regulating Factors (GRF) are a family of plant-specific transcription factors (TF) that have roles in plant growth, development and stress response [1]. The first *GRF* gene was characterized in rice as a factor involved in stem elongation under flooding or in response to gibberellic acid [2]. Later the *GRF* TF genes were identified in the model plant species *Arabidopsis thaliana*, as well as a number of agricultural crops [1,3,4]. These genes are mainly expressed in young growing tissues and organs [1,3,5].

The protein sequence of the GRF TF contains two conserved domains, QLQ (Gln, Leu, Gln) and WRC (Trp, Arg, Cys) in the N-terminus. The C-terminal region of GRF proteins is variable and may have transcriptional activation activity. The WRC domain consists of a nuclear localization signal and a DNA-binding motif and is involved in binding to cis-acting regions of gene promoters [2,3]. The QLQ domain is involved in interaction with GRF-interacting factor proteins (GIF) [6]. GIF proteins through their SNH (SYT N-terminal homology) domains interact with GRF proteins to form functional complexes that participate in regulation of expression of downstream genes [5]. DELLA proteins, accumulation of which is characteristic of green revolution varieties, can interfere with OsGRF4-GIF1 interaction [7].

The level of *GRF* gene expression is in part regulated by miRNA396 in the post-transcriptional stage. Thus, *GRF* genes participate in regulation of the same growth, development and stress-response processes as miRNA396 [8].

GRF TFs are involved not only in gibberellin, but also in other plant hormone signaling pathways. Auxin-response factors regulates expression of some *GRF* genes, like *AtGRF5* and *AtGRF6* in *Arabidopsis*, that lack miRNA396 regulation [9]. On the other hand, upregulation of *OsGRF6* in rice plants, having the miRNA396 blocked by target mimicry, activates auxin biosynthesis and signaling, stimulating development of auxiliary branches and spikelets in panicle [10]. Enhanced expression of *OsGRF4* in rice activates brassinosteroid-responsive genes that enhances the growth of seedlings and source leaves, and promotes grain development. Enhanced brassinosteroid responsiveness may in turn change the level of gibberellins in plants [11]. 

In *Arabidopsis, GRF* genes positively regulate leaf growth and are involved in regulation of the stress response to heat, drought, salinity and diseases [3,12]. In rice higher expression of *OsGRF4* was shown to be connected to higher 1000 kernel weight and grain yield [11,13], higher nitrogen uptake and assimilation in plants carrying the *Slr1* gibberellin-insensitive reduced height gene [7]. In rapeseed *GRF2* was found to enhance seed oil production by increasing leaf area and photosynthetic efficiency [4]. Some rare alleles of *TtGRF4-A* (a homolog of *TaGRF9-6A*) associated with increased grain weight were found in wild emmer wheat [14]. Thus, gain-of-function mutations of the *GRF* genes have great potential for increasing yields of agricultural crops through increasing the leaf area, the size of fruit organs and nutrient use efficiency [15].

In bread wheat (*Triticum aestivum* L.) 30 *TaGRF* genes were phylogenetically divided into four groups. They were designated using numbers from 1 to 12, each number being unique for a group of homeologs, and a chromosome name [1]. These genes are highly expressed in growing tissues including stem meristem and reproductive organs [1]. The level of expression of the *TaGRF* genes changes significantly under osmotic, drought or salt stress [1,5].

In this study we investigated the TraesCS2A02G435100 gene of bread wheat, which is *TaGRF3-2A* according to the nomenclature of Huang et al. (2021), and is known as *TaGRF3* according to Zan et al. (2020) [1,5]. This gene was chosen as one of the most similar to the rice *OsGRF4* gene with the highest percentage of query cover in BLAST output (however the most similar to *OsGRF4* are *TaGRF9-6A, -6B* and *-6D*). The *TaGRF3-2A* gene is primarily expressed in shoot apical meristem, stigma and ovary, seeds and young leaves. Also, it seems to be responsive to phosphorous starvation and drought stress [1].

## 2. Results

### 2.1. TaGRF3-2A Alleles

As a result of the next-generation sequencing of the polymerase-chain-rection-amplicons of the *TaGRF3-2A* gene (TraesCS2A01G435100) and its flanking regions in 19 winter bread wheat cultivars (Table S1), and comparing them with the genome sequences of 13 more varieties that were included in the Wheat 10+ Genomes Project [16] and the genome of the Chinese Spring bread wheat [17] (a total of 33 varieties of bread wheat), we found a total of 21 haplotypes. The considered region on wheat chromosome 2A covered 1,075 nucleotides before the start codon, and 1,389 nucleotides after the stop codon of the *TaGRF3-2A* gene, completely capturing the 5′ and 3′ untranslated regions (UTR) and part of the promoter. Also, in spelt wheat PI428198 (*Triticum spelta* L.), wild emmer wheat Zavitan (*Triticum dicoccoides* (Koern. ex Aschers. Et Graebn.) Schweinf.) and diploid wheat (*Triticum urartu* Thum. ex Gandil.), that were included in the comparison, three more haplotypes were found [16,18,19]. The *T. urartu* haplotype differed from the bread wheat haplotypes in multiple unique single-nucleotide variants, the haplotype of wild emmer had fewer differences, and the spelt haplotype did not have unique point mutations and differed only by a combination of the highly variable single nucleotide polymorphism (SNP) and the length of a microsatellite in the 5′UTR.

In the promoter of the gene, we found a frequently occurring polymorphism chr2A:g.687048627G>C (sequence variations are described according to HGVS recommendations, IWGSC RefSeq v1.0 wheat genome is used as reference) [20]. At this position, most varieties of bread wheat, as well as its wild relatives included in our study, have nucleotide variant ‘C′, whereas wheat accessions Chinese Spring, Jagger, Lancer, CDC Stanley, Norin 61 and Novosibirskaya 67 have variant ‘G′. Analysis of the promotor sequence using the PlantPAN 3.0 database showed that variant ‘G′ gives an additional basic helix-loop-helix transcription factor binding site to the promoter. Several minor frequency SNPs were found in the promoter: an alternative variant of the polymorphism chr2A:g.687048382C>T is present in cultivars Grom and Altigo, variant ‘T′ of the polymorphism chr2A:g.687048137G>T is present in varieties Velena and Paragon. Variant ‘G′ of the polymorphism chr2A:g.687048328A>G is present only in *T. urartu* (PI428198) and *T. dicoccoides* (Zavitan). In the considered region of the promoter (chr2A:g.687047878_687048697) we found 15 unique single-nucleotide variants in the *T. urartu* haplotype.

**Figure 1:**
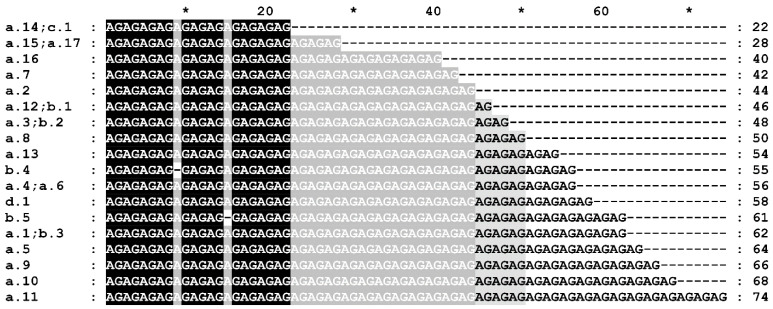
Microsatellite length variation in the 5′UTR of the *GRF3-2A* gene detected by sequencing. The *GRF3-2A* haplotypes are indicated in the left. An asterisk (*) in the upper line marks each 10-th nucleotide in the alignment between the numbers.

In the 5′UTR of the *Grf3-2A* gene, there is a microsatellite repeat (AG)_n_, where the number of repeats *n* varies from 11 to 37. Some variants of the microsatellite differ in loss of a single nucleotide A, resulting in a repeat length of 55 and 61 nucleotides (Figure 1). The wild emmer wheat Zavitan has a deletion of 12 nucleotides c.-102_-91del at a short distance before the microsatellite, and also a single nucleotide substitution c.-140A>G in the 5′UTR.

*T. urartu* PI428198 has two single-nucleotide substitutions c.*285A>G and c.*312A>T in the 3′UTR, and the bread wheat CDC Landmark has a substitution c.*8C>T.

In the 3′-end flanking region immediately after the transcription stop site, *T. urartu* PI428198 has a single nucleotide substitution chr2:g.687052487C>T, and the wild emmer Zavitan has a deletion of two nucleotides at the same point—chr2:g.687052487_687052488del.

Within the introns of the gene, differences from the reference Chinese Spring genome (IWGSC RefSeq v1.0) were found only in *T. urartu*. *T. urartu* accession PI428198 has an insertion of three nucleotides c.325+32_+33insTCC, as well as three single-nucleotide substitutions, c.325+169G>C, c.326-39A>G, and c.326-174A>G, in the second intron. Single-nucleotide substitutions c.689+136A>G and c.689+241T>C were found in the third intron.

In the protein-coding sequence of the *GRF3-2A* gene, a deletion of nine nucleotides c.126_134del was detected in the second exon in Novosibirskaya 67. This mutation led to the loss of three glutamine residues in the protein molecule in the polyglutamine region p.(Gln42_Gln44del). In the third exon, we found a c.495G>T polymorphism that is frequent among Russian winter wheat cultivars, leading to the replacement of amino acid residue glutamine with histidine p.(Gln165His). In the third exon of the *T. urartu* gene there are two adjacent single-nucleotide substitutions relative to the reference genome—c.528G>C and c.530C>G, which should result in the replacement of two consecutive amino acids in the protein p.(Gln176_Ala177delinsHisGly). In the 4th exon, *T. urartu* also has a c.729C>G missense variant, which results in a p.(Asp243Glu) amino-acid substitution.

### 2.2. Protein Isoforms

Following the above-described amino acid substitutions, 4 isoforms of the GRF3-2A protein were found among the examined wheat accessions. According to the frequencies of occurrence, we designated them as follows: A—the most frequent isoform, presents in Chinese Spring and most other cultivars, B—frequently found among Russian winter cultivars, C—the isoform characteristic of *T. urartu*, and D—a mutant form found only in Novosibirskaya 67 (Figure S1). Among accessions in which the *TaGRF3-2A* was completely sequenced, isoform B was present mainly among cultivars developed at Krasnodar. The correspondence of isoforms to amino acid substitutions, as well as the estimated significance of the amino acid changes for biological function of the protein, predicted by PROVEAN, are shown in Table 1. Isoform B, according to PROVEAN, has a functionally significant amino acid substitution. However, no mutation found in this study disturbed the WRC or QLQ conserved domains of the protein (Figure S1).

### 2.3. Phylogenetics of Protein Sequences

**Figure 2:**
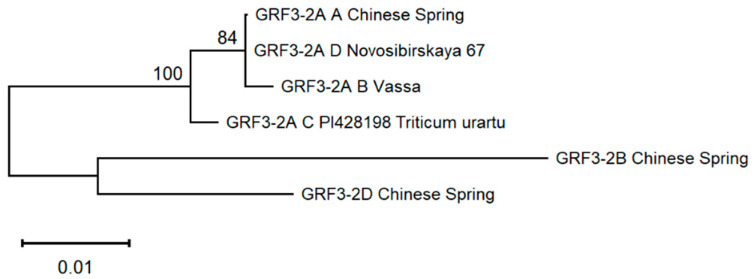
Molecular phylogenetic tree of GRF3-2A protein isoforms. GRF3-2B and GRF3-2D proteins of Chinese Spring were taken as outgroups.

Phylogenetic analysis of the predicted amino-acid sequences of the GRF3-2A protein showed that isoform C characteristic of *T. urartu* is the most ancient, whereas isoform B is the newest (Figure 2). Isoform D, unique for Novosibirskaya 67, is a bit closer to the root of the tree than isoform A, apparently due to the proteins GRF3-2B and GRF3-2D, used as outgroup, having a smaller number of consecutive glutamine residues than GRF3-2A (5 vs. 7, beginning from 38 or 39 residue). Isoform D has 4 glutamine residues in that part of the molecule (Figure S1).

### 2.4. Allele Designation

For further discussion, we designated the various haplotypes (alleles) of the *GRF3-2A* gene together with its flanking regions with small letters in accordance with the encoded protein isoforms (*a*, *b*, *c*, *d*) and the number after a dot assigned in accordance with the frequency of occurrence (1—for the most frequent, 2, 3, etc.—for rarer haplotypes) within each group of haplotypes encoding the same protein. The sequences of all haplotypes are represented in Supplementary Materials in FASTA format, the wheat accessions in which they were found are listed in Table S2, the frequency of occurrence among the sequenced accessions is listed in Table S3.

### 2.5. Allele Phylogenetic Analysis

**Figure 3:**
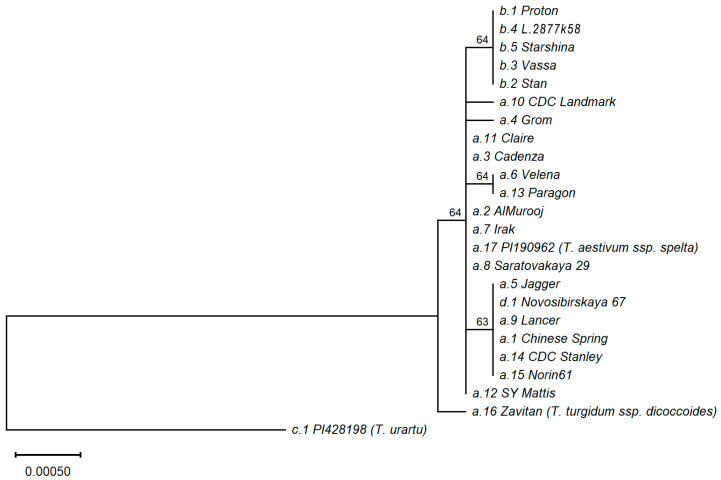
Molecular phylogenetic tree (subtree) of *GRF3-2A* haplotypes (gene + flanking sequences). To find a root, *GRF3-2B* gene of Chinese Spring was taken as an outgroup (not shown here). Bootstrap values are shown above nodes. At the tree leaves the *Grf3-2A* haplotypes and representative wheat accessions are indicated. If not specified, the accessions are *Triticum aestivum* L. ssp. *aestivum*.

The phylogenetic analysis of the *Grf3-2A* and flanking region haplotypes was carried out using the SNPs only. Based on the alignment of the nucleotide sequences and constructed phylogenetic tree, it is clear that the *T. urartu* (PI428198) haplotype is evolutionarily distant from all others (Figure 3). The haplotype of wild emmer *T. dicoccoides* (Zavitan) was much closer to the haplotypes of bread wheat, although it has significant differences from them. Bread wheat haplotypes were divided into six groups. The older group includes *T. spelta* (haplotype *a.17*), as well as haplotypes *a.2*, *a.3*, *a.8*, *a.11* and *a.12* of bread wheat. There were also five younger groups: (1) a group that includes haplotypes of Chinese Spring (*a.1*) and Novosibirskaya 67 (*d.1*); (2) a group that includes all *b* haplotypes (*b.1 … b.5*); (3) a group that includes haplotypes *a.6* (Velena) and *a.13* (Paragon); (4) haplotype *a.10* (CDC Landmark); (5) haplotype *a.4* (Grom). The last two groups include only one haplotype. Judging by the alignment of nucleotide sequences, the differences between haplotypes within the same group are due either to the presence of indels (in case of *d.1*, *c.1* and *a.16*), or to a change in the length of the microsatellite in the 5′UTR.

### 2.6. Molecular Markers and Phenotype

**Figure 4:**
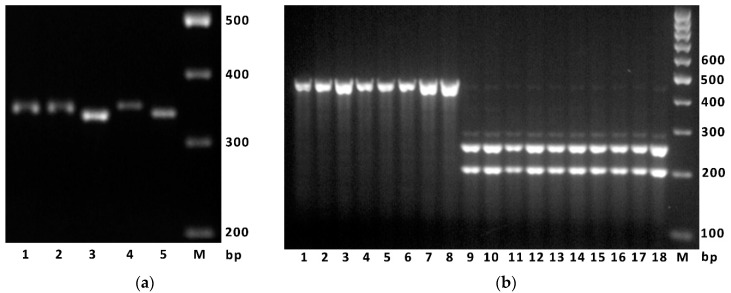
(**a**) An example of electrophoresis of the PCR marker designed for detecting the c.126_134del polymorphism. The PCR products were obtained using the primers GRF3A-Q42-F/R and DNA of the following wheat accessions: lane 1—Vid, 2—Stan, 4—Altigo, 3 and 5—Novosibirskaya 67, M—size standard M-100 (Syntol LLC, Moscow). (**b**) An example of electrophoresis of the marker detecting the c.495G>T polymorphism in *GRF3-2A*. The PCR products obtained using primers GRF3A-Q165-F/R and digested using *Sfa*N I endonuclease. Lanes 1–8—accessions having the *GRF3-2Aa* allele (nucleotide G), two lanes each: Grom, Altigo, Velena, Sila; lanes 9–18—accessions having the *GRF3-2Ab* allele (nucleotide T), two lanes each: Vassa, Proton, Alekseich, Vid, Stan. M—size standard M-100.

We designed subgenome-specific primers to detect the 9-nucleotide deletion in the second exon of the *GRF3-2A* gene that was found in Novosibirskaya 67 (see Section 4). The PCR with DNA of the Novosibirskaya 67 gave a fragment of 335 base pairs (bp) of expected size (Figure 4a). Screening of 199 winter bread wheat accessions from a collection of the National Center of Grain (Krasnodar, Table S4) showed that among them there was only the 344 bp variant of the marker. This suggests that the Novosibirskaya 67 cultivar carries a rare mutation of the *GRF3-2A* gene.

A marker designed for detection of the c.495G>T mutation resulting in amino-acid substitution, p.(Gln165His), was successfully validated on the DNA of wheat accession in which the *GRF3-2A* gene was sequenced (Figure 4b).

When a pool of 199 winter bread wheat accessions was analyzed using this marker, we found that the variant T of the polymorphism c.495G>T inherent to *GRF3-2Ab* allele was present in almost 39% of accessions (Table S5). *GRF3-2Ab,* found in Bezostaya 1 and Krasnodarskaya 6 (old Russian cultivars), was in 46% of modern Russian cultivars, and was also detected in some Bulgarian, Chinese, Polish, Romanian, Ukrainian, USA and Yugoslavian cultivars, most of which, but not all, have Bezostaya 1 in their pedigrees. Cultivars from Austria, the Czech Republic, France, Germany, Hungary, and the UK that were tested did not have *GRF3-2Ab*. Due to low numbers of accessions from countries other than Russia, we cannot assert statistically significant differences of allele frequencies between different geographical locations.

**Figure 5:**
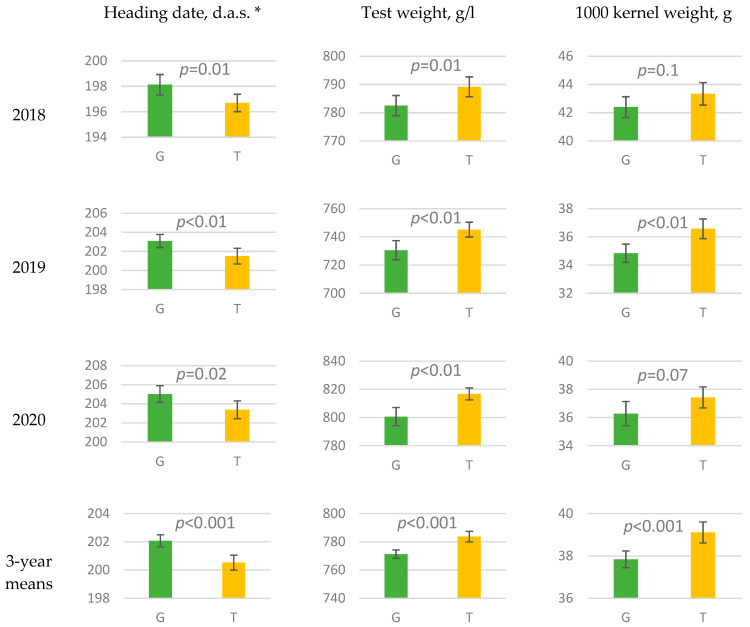
Heading date and grain traits for winter wheat accessions differing in missense mutation c.495G>T in *TaGRF3-2A* tested in 2018–2020 at Krasnodar. Bars indicate 95% confidence intervals. The *p*-values are calculated for Fisher′s F-test. *—days after sowing.

Statistical analysis of phenotypic data for 199 winter bread wheat accessions collected over three years (2018–2020) at Krasnodar showed that the *GRF3-2Ab* allele (T variant at c.495G>T polymorphism) in each of the three years was significantly associated with earlier heading (*p* ≤ 0.02, Fisher′s F-test) and higher test weight of the grain (*p* ≤ 0.01) compared to the *GRF3-2Aa* allele (Figure 5). In one year, a positive association of the *GRF3-2Ab* allele with 1000 kernel weight (2019, *p* < 0.01) and a high grain protein content (2018, *p* = 0.01) was revealed. However, in 2018, the grain yield was significantly lower for winter wheat accessions with the *GRF3-2Ab* allele, which presumably was associated with lower either grain numbers per spike or tillers per plant. However, the protein yield per hectare was non-significantly decreased in accessions carrying *GRF3-2Ab* in 2018 (Figure S2). Mean values of agronomic traits among wheat accessions having different alleles of the c.495G>T polymorphism are represented in Table S7.

Comparison of the 5′UTR microsatellite among sequenced *GRF3-2A* haplotypes and haplotypes obtained from sequenced wheat genomes gave 18 different lengths of the tandem repeat (Figure 1). However, the GRF3-2AD-SSR marker tested on the 199 winter wheat accessions showed only nine of those variants. That could be explained by the different accessions that were sequenced and genotyped. Statistical analysis showed significant connection between the microsatellite marker and the agronomic traits (Figures S3 and S4). Mean values of agronomic traits among wheat accessions having different alleles of the microsatellite are represented in Table S8. However, the same microsatellite size was observed for different gene alleles, including those that code different protein isoforms (Figure 1).

## 3. Discussion

As expected for a functional gene prone to natural selection, most of the polymorphisms in *GRF3-2A* were detected in the non-coding regions and flanking sequences, while coding sequences were more conserved. 

The *GRF3-2A* haplotypes of ancestral wild wheat species were evolutionary distant from the haplotypes of the bread wheat cultivars, indicating that wild species might serve as a source for the new alleles for investigation and crop improvement.

Although the observed polymorphisms in the promotor, 5′ and 3′UTR do not change the protein sequence, they could alter the level of the RNA transcripts and translated protein. Mutations in the promoter can alter the number and composition of the cis-acting elements, recognized by transcription factors [21], while mutations of the DNA sequence in the region of the polyadenylation signal can affect the length of the mRNA and the presence of targets for microRNA and other post-transcriptional regulation factors in it [22]. The length of the 5′UTR microsatellite could also influence the level of gene expression [23].

Spring bread wheat cultivar Novosibirskaya 67 had a rare mutation that was not reported for this gene before—a deletion of nine nucleotides in the second exon, leading to a deletion of three glutamine residues from the protein molecule. We designated this mutation as *TaGRF3-2Ad,* corresponding to protein isoform D. The PROVEAN prediction, based on the change in alignment score of the amino-acid sequence to itself and to other related sequences in the database, showed that the biological function of the GRF3-2A protein should not be significantly altered by this mutation. Novosibirskaya 67 was one of the most widely grown spring wheat cultivars in Siberia since 1974 until the end of the 20th century. It was bred from a population of mutants obtained from the cultivar Novosibirskaya 7 following radiation [24]. We did not find the *TaGRF3-2Ad* allele in any other wheat cultivar, thus we assume that this mutation resulted from artificial mutagenesis, and may confer some adaptive traits to Novosibirskaya 67. The agronomic value of the *TaGRF3-2Ad* allele would become the subject of further study.

The c.495G>T missense mutation found in our study was present in many Russian winter wheat cultivars. We designated the allele carrying this mutation as *TaGRF3-2Ab*. It is present in almost half of the modern cultivars developed at the National Center of Grain at Krasnodar and is present in the old cultivars Bezostaya 1 and Krasnodarskaya 6. Previously, this mutation was reported in the results of the 1000 wheat exomes project and mapped to the reference wheat genome (IWGSC RefSeq v1.0) at coordinate chr2A:687050412 [25]. Among 811 wheat accessions tested in that project, the c.495G>T missense mutation was found in only 3% of genotypes, including cultivars from the former USSR, Bulgaria, Argentina, Mexico, and some other countries.

Statistical analysis of the agronomic traits tested in 199 wheat accessions grown in Krasnodar during the three years showed that the *TaGRF3-2Ab* allele is associated with earlier heading and better grain filling as judged by the test weight and 1000 kernel weight. However, in some years *TaGRF3-2Ab* was associated with lower grain yield per hectare. In separate years, as in 2018, the mean yield of the cultivars and lines carrying *GRF3-2Ab* was even lower than in those carrying *GRF3-2Aa*. Most likely, *GRF3-2Ab* acts through the restriction of additional tillering at the late development stages of the plant, which releases the resources of assimilates for grain formation. This assumption has not been tested directly yet, but it can explain the observations. For winter wheat that may be exposed to severe winter conditions or even to drought in spring, which may become more often due to global warming, additional tillering may be favorable for the recovery of the crop. Although *GRF3-2Ab* may be good at maintaining the high milling quality of the grain, we think it should be used with caution in breeding of winter wheat cultivars from the perspective of climate change.

The *TaPPD1-2A* gene (TraesCS2A02G081900) in chromosome 2A that can affect earliness [26] is located at a considerable physical distance from *TaGRF3-2A*, probably on the other chromosome arm. Thus, the early heading associated with the *TaGRF3-2Ab* allele is unlikely to be explained by genetic linkage with the *PPD1* gene. Other genes that could affect earliness, such as *Vrn-1* (*Vernalization*) or *Eps-1* (*Earliness per se*) are located on other chromosomes [27,28]. However, the presence of a nearby gene linked to *TaGRF3-2A* and affecting earliness cannot be excluded. We screened numerous genome-wide association studies reporting SNP markers connected with agronomic traits, and found no one significant marker-trait association that hit exactly the locus of the *TaGRF3-2A*. However, we found two markers associated with heading time—BobWhite_c16923_64 and wsnp_Ex_c14953_23104041—which surround the *TaGRF3-2A* and are 128 and 42 mega base pairs (Mbp) distant from it correspondingly [29]. Also, a marker BS00009247 on chromosome 2B located 11 Mbp from *TaGRF3-2B*—a homeolog of the gene studied—was associated with heading time [30]. Further fine mapping and gene engineering experiments are needed to postulate the causal relationship between *TaGRF3-2A* and the phenotype.

Breeding for early maturity and plump grain, together with the initial germplasm used, can explain the high frequency of *TaGRF3-2Ab* in Russian cultivars. Bezostaya 1 is present in pedigrees of the most modern Russian winter wheat cultivars, and thus is the probable source of the *TaGRF3-2Ab* allele in them. Bezostaya 1 probably obtained this allele from the old cultivar Ukrainka [25], or from Krymka (a Crimean landrace) [31,32] both of which are in the pedigree of Bezostaya 1 and other cultivars carrying *TaGRF3-2Ab,* but not having Bezostaya 1 or Ukrainka in pedigree. The presence of the *TaGRF3-2Ab* in Krymka could explain its presence in some American (USA) cultivars [33]. Generally, we can assume that *TaGRF3-2Ab* was present in multiple local cultivars (landraces) grown in lands surrounding the Black Sea before the beginning of the scientific breeding. 

## 4. Materials and Methods

### 4.1. Plant Material and Phenotyping

Most of the wheat accessions used for sequencing *GRF3-2A*, genotyping and phenotyping were a part of the winter bread wheat collection maintained at the National Center of Grain named after P. P. Lukyanenko in Krasnodar (Table S4). Spring wheat accessions from Iraq were provided by Dr. Oleg G. Semenov (Department of Technosphere Safety, Agrarian-Technological Institute, RUDN University, Moscow). Other spring wheat accessions used for DNA extraction, PCR and sequencing were a part of a mini-collection maintained by the authors in Moscow (Table S1).

Yield testing and phenotyping for other parameters of the 199 winter wheat accessions was conducted according to the “Competitive variety testing methodologies” during the 2018–2020 harvest years at Krasnodar [34]. The experiments were laid in randomized complete block design on 25 m^2^ field plots in 4 replications. 1000 kernel weight was determined using a SEED COUNTER S-25 device (DATA Detection Technologies Ltd., Kibbutz Tzora, Israel) and electronic scales. Test weight, grain moisture content, and grain protein content were measured on 700 g samples using an Infratec 1241 grain analyzer (FOSS, Hilleroed, Denmark). Grain yield was determined as grain mass at 14% moisture content divided by plot area excluding the area of damaged sites. The heading date was recorded when 75% of plants showed at least a 50% spike emergence from the flag leaf sheath. Mean values of the measurements in each year were used for statistics regarding molecular markers.

### 4.2. Weather Conditions

The weather conditions during the vegetation periods in 2017–2020 for winter wheat at Krasnodar are represented in the Figures S5 and S6. The weather data were provided by the nearest meteorological station. The sowing of winter wheat was done on 21 October (both in 2017 and in 2018), and on 17 October in 2020. All three crop years were characterized by a dry period during seedbed preparation at the beginning of autumn and warm winters with no hard frosts and low precipitation, during which the plants maintained slow growth. The booting stage was observed in the last third of March, while heading and anthesis occurred at the beginning of May in each year. In 2018 March was wet, and from April to the end of vegetation the precipitations were lower than average. This resulted in partial loss of young tillers in 2018. The spring of the 2019 was close to the climatic average. March and April of the 2020 were characterized by severe rainfall deficiency and recurrent frosts that caused partial loss of the main stems of early-maturing lines, while in May rainfall was higher than average. Grain filling and maturation during all three years occurred under conditions of high temperature and low humidity.

### 4.3. DNA Extraction, PCR and Sequencing

Genomic DNA was extracted from dried ground leaves of seedlings using the CTAB protocol [35]. The sequence of *TaGRF3-2A* (TraesCS2A02G435100) with about 1000 base pairs (bp) of flanking sequence, as well as the sequences of its homeologs on chromosome 2B and 2D (TraesCS2B02G458400 and TraesCS2D02G435200), were obtained from the annotated wheat genome assembly IWGSC RefSeq v1.0 using genome browser [17]. Specific primer pairs giving overlapping PCR products were designed using Primer-BLAST (NCBI) [36] to amplify the entire *TaGRF3-2A* sequence with a 1000 bp promotor region (Table 2). The specificity of the primers was rechecked using alignment of the three homoeologous genes.

PCR was performed in 25 μL reaction volumes, containing 70 mM Tris–HCl buffer (pH 9.3), 16.6 mM (NH_4_)_2_SO_4_, 2.5 mM MgCl_2_, 0.2 mM of each dNTP, 0.3 μM forward and reverse primers (Sintol Ltd., Moscow, Russia), 0.04 U/µL LR (long reading) Plus polymerase (Sileks Ltd., Moscow, Russia), 0.02 U/µL Taq polymerase (Sileks Ltd.), and 4 ng/µL DNA template. PCR conditions were as follows: (1) 95 °C for 10 min, (2) 45 cycles of 95 °C for 30 s, 60 °C for 30 s, 72 °C for 4 min; and (3) final extension step of 72 °C for 10 min. PCR products were separated in 1.5% agarose gels in TBE (90 mM Tris, pH 8.3, 90 mM boric acid, 0.1 mM EDTA) buffer using GeneRuler 100 bp Plus DNA Ladder (Thermo Fisher Scientific, Waltham, MA, USA) as a molecular weight marker, and stained with ethidium bromide for subsequent visualization in Gel Doc XR+ (Bio-Rad Laboratories, Inc., Hercules, CA, USA).

In cases of successful amplification, the PCR products obtained from DNA of the same wheat plant were mixed and submitted for NGS sequencing on Illumina MiSeq system. Sequencing was performed at “Genomed, Ltd.” (Moscow, Russia). DNA libraries were prepared using Swift 2S™ Turbo DNA Library Kits. In the process of library preparation, the contents of each tube, corresponding to a single wheat plant, were labelled with individual DNA barcodes. The gene sequences for each wheat plant were reconstructed using the previously published algorithm [37,38]. The 19 wheat accessions in which the *TaGRF3-2A* gene was sequenced in this way are listed in Table S1.

### 4.4. Sequences Obtained from Genome Assemblies

*GRF3-2A* sequences were obtained from the assembled genomes of bread wheat [16], durum wheat [39], spelt [16], *T. urartu* [19] and wild emmer [18] wheat (Table S6). The rough coordinates of *GRF3-2A* sequences were found in the genomes using the BLAST+ command line tool [40]. Using these coordinates, the sequences with extended margins were extracted from the genome FASTA files using a program written in Python [41].

### 4.5. Sequence Analysis and Phylogenetics

The sequences of *TaGRF3-2A* gene obtained experimentally along with others found in assemblies of wheat genomes were aligned using the MUSCLE algorithm in MEGA X software [42,43]. The exons and protein-coding sequences were detected using alignment with such sequences annotated for TraesCS2A02G435100 in IWGSC RefSeq v1.0. The translation of coding DNA sequence to an amino acid sequence was performed in GeneDoc 2.7 software [44].

Protein domains were identified using a Conserved Domains Database search at the NCBI website [45]. The functional significance of amino acid substitutions was predicted using the PROVEAN online service [46].

Evolutionary analyses of the *TaGRF3-2A* DNA sequences including a 1000 bp promotor were conducted in MEGA X software using the maximum-likelihood method and Hasegawa–Kishino–Yano model [47]. All positions containing gaps and missing data were eliminated. The evolutionary tree of protein sequences was constructed using the maximum-likelihood method and Jones-Taylor-Thornton (JTT) model [48]. All sites, including gaps, were used. For both DNA and protein phylogenetic analyses, bootstrap support values were calculated using 500 replicates. The trees were drawn to scale, with branch lengths measured by numbers of substitutions per site. To establish a tree root, the homologs from wheat subgenome B and D were added as outgroups.

The analysis of the promoter sequence for the presence of transcription factor binding sites was done using the PlantPAN 3.0 database [21].

### 4.6. Pedigrees

The pedigrees of the wheat accessions studied were obtained from the Genetic Resources Information System for Wheat and Triticale website [49], or from the website of the State Commission for Selection Achievements Test and Protection (Russia) [50].

### 4.7. Molecular Markers

For detection of the 9-nucleotide deletion c.126_134del resulting in deletion of the three amino-acid residues p.(Gln42_Gln44del) of the GRF3 protein, we designed a pair of primers giving PCR products of 335 or 344 base pairs: GRF3A-Q42-F: 5′-CTTCTATCTGTAGCTCGAGGTGT-3′ and GRF3A-Q42-R: 5′-GTGGTAGGAGGAGGAGGAATCTA-3′.

PCR was performed in 25 μL reaction volumes, containing 70 mM Tris–HCl buffer (pH 8.6), 16.6 mM (NH_4_)_2_SO_4_, 2.5 mM MgCl_2_, 0.2 mM of each dNTP, 0.3 μM forward and reverse primers (Sintol Ltd., Moscow, Russia), 0.05 U/µL Taq polymerase (Sileks Ltd., Moscow, Russia), 4 ng/µL DNA template. The PCR conditions were as follows: (1) 95 °C for 10 min, (2) 36 cycles of 95 °C for 30 s, 60 °C for 30 s, 72 °C for 1 min; and (3) final extension step of 72 °C for 10 min. PCR products were separated in 2% agarose gels with TBE buffer for at least 1 h in an electric field intensity of 6 V/cm and visualized as described above. 

To detect missense mutation c.495G>T leading to amino-acid change p.(Gln165His) we designed a pair of primers: GRF3A-Q165-F: 5′-GGGTTTTCTTAATTTGCTTGCAGT-3′, GRF3A-Q165-R: 5′-CAGAAGATAAAAACGGCAGGTGA-3′. The PCR conditions were as described above. The PCR product of 454 bp was subjected to endonuclease digestion using SfaN I enzyme (SibEnzyme Ltd., Novosibirsk, Russia) having recognition site GCATC(5/9)^. In a case of nucleotide T in the c.495G>T polymorphism (resulting in histidine in the protein chain), the PCR product were digested into 201 and 253 bp products, while in the case of nucleotide G, the PCR product remained intact. The products of digestion were separated into 1.5% agarose gels in a TBE buffer using GeneRuler 100 bp DNA Ladder (Thermo Fisher Scientific, Waltham, MA, USA) as a molecular weight marker, and stained with ethidium bromide for subsequent visualization in Gel Doc XR+ (Bio-Rad Laboratories, Inc., Hercules, CA, USA).

For detection of simple sequence repeat length polymorphism in the 5′UTR of the *TaGRF3-2A* gene we used primer pair GRF3-2AD-SSR-F: 5′-TCTCACCAGGCAGCAGATCG-3′ and GRF3-2AD-SSR-R: 5′-ACAGGGAGGCAAAGGGCATC-3′ which was also suited to the *TaGRF3-2D* gene. The reverse primer was 5′-labelled with 6-carboxyfluorescein. As predicted by gene sequencing, the length of the PCR products for the *TaGRF3-2A* gene was expected in range 230 to 282 bp, whereas for *TaGRF3-2D* the expected sizes were 211 to 223 bp [51]. Thus, the PCR-product sizes of *TaGRF3-2A* and *TaGRF3-2D* genes do not overlap. The PCR products were diluted 100 times and subjected to fragment analysis on a Nanofor-05 genetic analyzer (Sintol Ltd., Moscow) using a fluorescent fragment size standard SD-450 (Sintol Ltd.).

### 4.8. Statistical Analysis

Calculation of means, analysis of variance and confidence intervals were done in the Statistica 6.0 software package. Three-year means of agronomic traits for genotypes were calculated as least square means in two-way analysis of variance, where a year was one of the factors. Fisher′s exact test for score traits was performed in the R programming language [52].

## 5. Conclusions

We studied the allelic diversity of the *GRF3-2A* gene in bread wheat and compared bread wheat alleles with those in some wild ancestral species. The allele designated as *TaGRF3-2Ab* was rare in a world wheat collection, but was quite common among Russian winter wheat cultivars. This allele was associated with earlier heading and better grain filling, while keeping almost the same yield per hectare. We can assume that this allele is adaptive for the steppes of the Black Sea region. We discovered a unique mutation of *TaGRF3-2A* in spring wheat cultivar Novosibirskaya 67, the agronomic value of which is yet to be established.

## Figures and Tables

**Table 1:** GRF3-2A protein isoforms and functional significance of amino acid differences.

Isoform.	Differences in Protein Sequence	PROVEAN Score	Representative Accessions *
A	-	-	Chinese Spring
B	Gln165His	−2.601 **	Stan, Vassa, Vid
C	Gln176_Ala177delinsHisGly	−1.224	PI428198 (*T. urartu*)
	Asp243Glu	0.524	
D	Gln42_Gln44del	0.821	Novosibirskaya 67

* If not specified, the species is *Triticum aestivum* L. ** Variants that have a score lower than −2.5 are assumed to be deleterious in protein biological function.

**Table 2:** Primers used for PCR-amplification of the *TaGRF3-2A* fragments.

Primer Sequence, 5′→3′	Tm, °C	Expected Product Size, bp
*GRF-2A*-1F: AAATTGAAGGCTAGACAATCGGC *GRF-2A*-1R: CCTTTTACTCCTACTTGCCTGGT	60	1179
*GRF-2A*-2F: CAAACGAACTTGACGGTACAGAT *GRF-2A*-2R: CACATGAGGATGAGGCTTCTTGA	60	1188
*GRF-2A*-3F: AGATTTCAGGTGTACTCGACCTC *GRF-2A*-3R: AGCATGCAGAAGATAAAAACGGC	60	1185
*GRF-2A*-4F: GCTCAGCTGCACATGGATAATG *GRF-2A*-4R: CGAGTCAGATTTGCAGCATAGTG	60	1119
*GRF-2A*-5F: TGCAGCAACAATTGCTCGTATAG *GRF-2A*-5R: CACCCCCACCCCTAAGATAGATA	60	1250

## Data Availability

The data presented in this study are available in Supplementary Materials to this article. The sequences of the *TaGRF3-2A* gene obtained in this study were deposited to NCBI GenBank under accession numbers OK094721-OK094732.

## Supplementary Materials

The following are available online at https://www.mdpi.com/article/10.3390/ijms222212376/s1.
