# Supplementary material for: Alleles of the GRF3-2A Gene in Wheat and Their Agronomic Value
Source: Int J Mol Sci. 2021 Nov 16;22(22):12376. doi: 10.3390/ijms222212376 (PMC8622619; doi:10.3390/ijms222212376)
Supplement: Supplementary file 1 [file ijms-22-12376-s001.zip › Figure S1 GRF3 Protein isoforms.pdf]

```
                      *        20         *        40         *        60         *        80         *        10
GRF3-2A_A : MAMPFASLSPAADHHRSSPIFPFCRSSPLYSAGEEAAQQQQQQQHAMSGARWAAARPATFTAAQYEELEQQALIYKYLVAGVPVPPDLLLPIRRGFDSL :  99
GRF3-2A_B : MAMPFASLSPAADHHRSSPIFPFCRSSPLYSAGEEAAQQQQQQQHAMSGARWAAARPATFTAAQYEELEQQALIYKYLVAGVPVPPDLLLPIRRGFDSL :  99
GRF3-2A_C : MAMPFASLSPAADHHRSSPIFPFCRSSPLYSAGEEAAQQQQQQQHAMSGARWAAARPATFTAAQYEELEQQALIYKYLVAGVPVPPDLLLPIRRGFDSL :  99
GRF3-2A_D : MAMPFASLSPAADHHRSSPIFPFCRSSPLYSAGEEAAQQ---QQHAMSGARWAAARPATFTAAQYEELEQQALIYKYLVAGVPVPPDLLLPIRRGFDSL :  96
GRF3-2B   : MAMPFASLSPAADHHRSSPIFPFCRSSPLYSVGEETAHQQQ-QQHTMSGARWAA-RPATFTAAQYEELEQQALIYKYLVAGVPVPPDLLLPIRRGFDSL :  97
GRF3-2D   : MAMPFASLSPAADHHRSSPIFPFCRSSPLYSAGEEAAQQQ--QQHAMSGARWAAMRPATFTAAQYQELEQQALIYKYLVAGVPVPPDLLLPIRRDFDSL :  97

            0         *       120         *       140         *       160         *       180         *        2
GRF3-2A_A : ASRFYHHHALGYGSYFGKKLDPEPGRCRRTDGKKWRCSKEAAQDSKYCERHMHRGRNRSRKPVETQLV------SHSQQLQQQAP---AAAFHGHSPYP : 189
GRF3-2A_B : ASRFYHHHALGYGSYFGKKLDPEPGRCRRTDGKKWRCSKEAAQDSKYCERHMHRGRNRSRKPVETHLV------SHSQQLQQQAP---AAAFHGHSPYP : 189
GRF3-2A_C : ASRFYHHHALGYGSYFGKKLDPEPGRCRRTDGKKWRCSKEAAQDSKYCERHMHRGRNRSRKPVETQLV------SHSQQLQQHGP---AAAFHGHSPYP : 189
GRF3-2A_D : ASRFYHHHALGYGSYFGKKLDPEPGRCRRTDGKKWRCSKEAAQDSKYCERHMHRGRNRSRKPVETQLV------SHSQQLQQQAP---AAAFHGHSPYP : 186
GRF3-2B   : ASRFYHHHALGYGSYFGKKLDPEPGRCRRTDGKKWRCSKEAAQDSKYCERHMHRGRNRSRKPVETQLVATPH-HSHSQQLQQHAPAASAAAFHSHSPYP : 195
GRF3-2D   : ASRFYHHHALGYGSYFGKKLDPEPGRCRRTDGKKWRCSKEAAQDSKYCERHMHRGRNRSRKPVETQLVATPHSHSHSQQLQQHAPAATAAAFHSHSPYP : 196

            00        *       220         *       240         *       260         *       280         *
GRF3-2A_A : AIATGAG--APGSFALGSTAQLHMDNAAAPYATAGAAGNKDFRYSAYGFRTSAMEDHNQFISAAMDTAMDNYSWRLLPAQNSSFSLSSYPMLSTLSDLD : 286
GRF3-2A_B : AIATGAG--APGSFALGSTAQLHMDNAAAPYATAGAAGNKDFRYSAYGFRTSAMEDHNQFISAAMDTAMDNYSWRLLPAQNSSFSLSSYPMLSTLSDLD : 286
GRF3-2A_C : AIATGAG--APGSFALGSTAQLHMDNAAAPYATAGAAGNKDFRYSAYGFRTSAMEEHNQFISAAMDTAMDNYSWRLLPAQNSSFSLSSYPMLSTLSDLD : 286
GRF3-2A_D : AIATGAG--APGSFALGSTAQLHMDNAAAPYATAGAAGNKDFRYSAYGFRTSAMEDHNQFISAAMDTAMDNYSWRLLPAQNSSFSLSSYPMLSTLSDLD : 283
GRF3-2B   : AIASGGG----GSFAVGS-AQLHMDNAASPYATAGAAGNKDFRYSAYGFRTSAMEEHNQFISAAMETAMENYSCRLMPAQNSSFSLASYPMLGTLGDLD : 289
GRF3-2D   : AIATGGGGAAGSFGLGS-AQLHMDNAAAPYATAGAAGNKDFRYSAYGFRTSALEEHNQFISAAMDTAMDNYSWRLMPAQNSAFSLSSYPMLGTLGDLD : 294

            300        *       320         *       340         *       360         *       380         *
GRF3-2A_A : QSAICSLAKTEREPLSFFGVGGGFDDDESAVKQENQTLRPFFDEWPKDRDSWPELQDHDSNHNNEAFSATKLSISIPVTSSDFSTTAGSRSPHGIYSR : 384
GRF3-2A_B : QSAICSLAKTEREPLSFFGVGGGFDDDESAVKQENQTLRPFFDEWPKDRDSWPELQDHDSNHNNEAFSATKLSISIPVTSSDFSTTAGSRSPHGIYSR : 384
GRF3-2A_C : QSAICSLAKTEREPLSFFGVGGGFDDDESAVKQENQTLRPFFDEWPKDRDSWPELQDHDSNHNNEAFSATKLSISIPVTSSDFSTTAGSRSPHGIYSR : 384
GRF3-2A_D : QSAICSLAKTEREPLSFFGVGGGFDDDESAVKQENQTLRPFFDEWPKDRDSWPELQDHDSNHNNEAFSATKLSISIPVTSSDFSTTAGSRSPHGIYSR : 381
GRF3-2B   : QSAICSLAKTEREPLSFFGGGGGFDDDDSAVKQENQTLRPFFDEWPKDRDSWPELQDHDANNSSNAFSATKLSISIPVTSSDFSTTAGSRSPNGIYSR : 387
GRF3-2D   : QSAICSLAKTEREPLSF-GGGGGFEDDESAVKQENQTLRPFFDEWPKDRDSWPELQDHDSNHNSNAFSATKLSISIPVTSSDFSTTAGSRSPHGIYSR : 391
```

Figure S1. Alignment of the GRF3-2A protein isoforms with GRF3-2B and GRF3-2D proteins. The WRC domain that functions in DNA binding is highlighted in green (PEPGRCRRTDGKKWRCSKEAAQDSKYCERHMHRGRNRSRKP), while the QLQ domain involved in protein-protein interactions is highlighted in yellow (FTAAQYEELEQQALIYKYLVAGVPVPPDLLLPI).
