# Supplementary material for: Alleles of the GRF3-2A Gene in Wheat and Their Agronomic Value
Source: Int J Mol Sci. 2021 Nov 16;22(22):12376. doi: 10.3390/ijms222212376 (PMC8622619; doi:10.3390/ijms222212376)
Supplement: Supplementary file 1 [file ijms-22-12376-s001.zip › Figure S2 Yield and grain protein content Q165H.pdf]

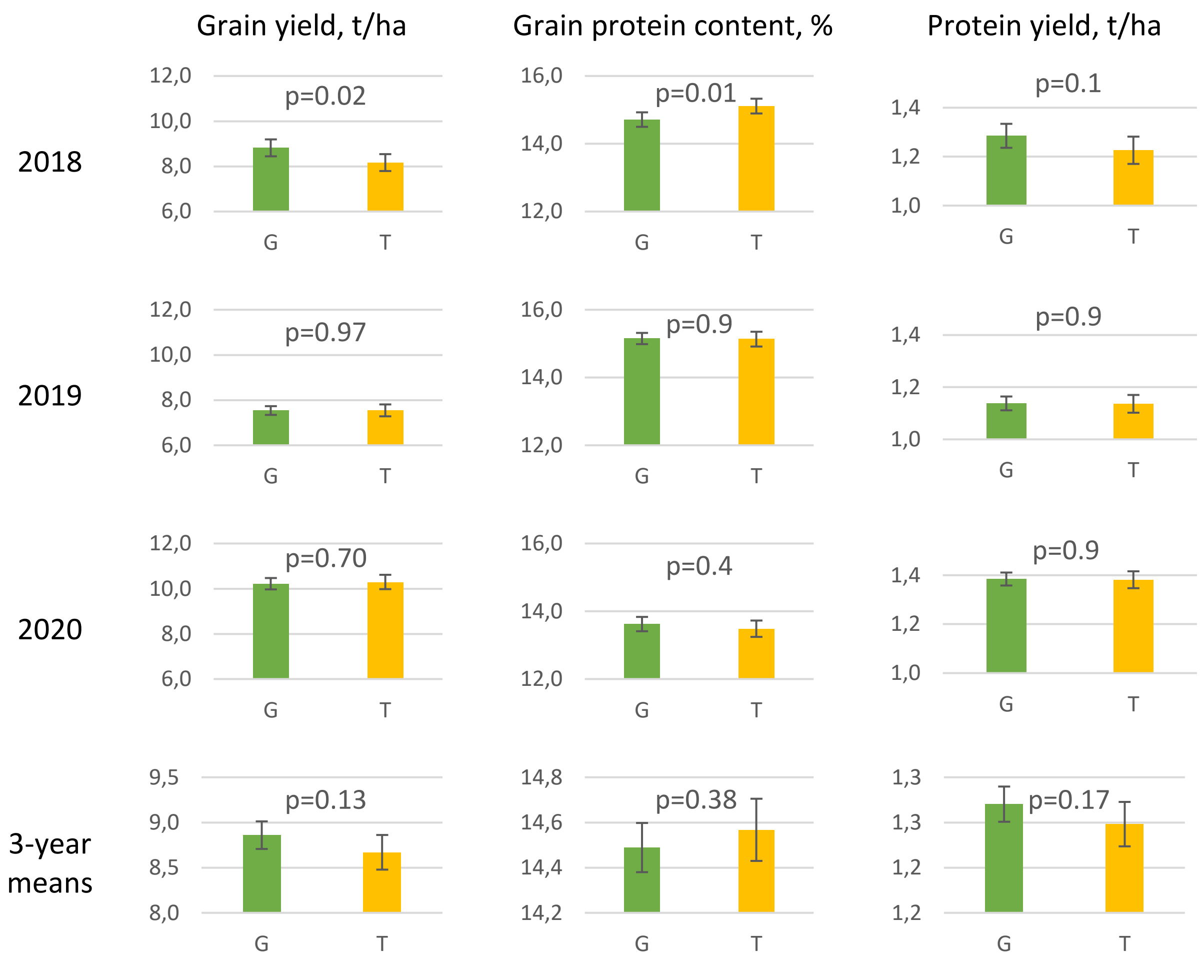

Figure S2. Grain yield, protein content and protein yield for wheat accessions differing in missense mutation c.495G>T in the *TaGRF3-2A* gene tested during 2018-2020 at Krasnodar. Bars indicate 95% confidence intervals. The p-values are calculated for Fisher's F-test. The percentages were transformed using $arcsin\left(\sqrt{x/100}\right)$ before ANOVA. The reverse-transformed means and boundaries of confidence intervals are shown.
