# Supplementary material for: Alleles of the GRF3-2A Gene in Wheat and Their Agronomic Value
Source: Int J Mol Sci. 2021 Nov 16;22(22):12376. doi: 10.3390/ijms222212376 (PMC8622619; doi:10.3390/ijms222212376)
Supplement: Supplementary file 1 [file ijms-22-12376-s001.zip › Figures S3-S4 Yield and grain traits 5-UTR SSR.pdf]

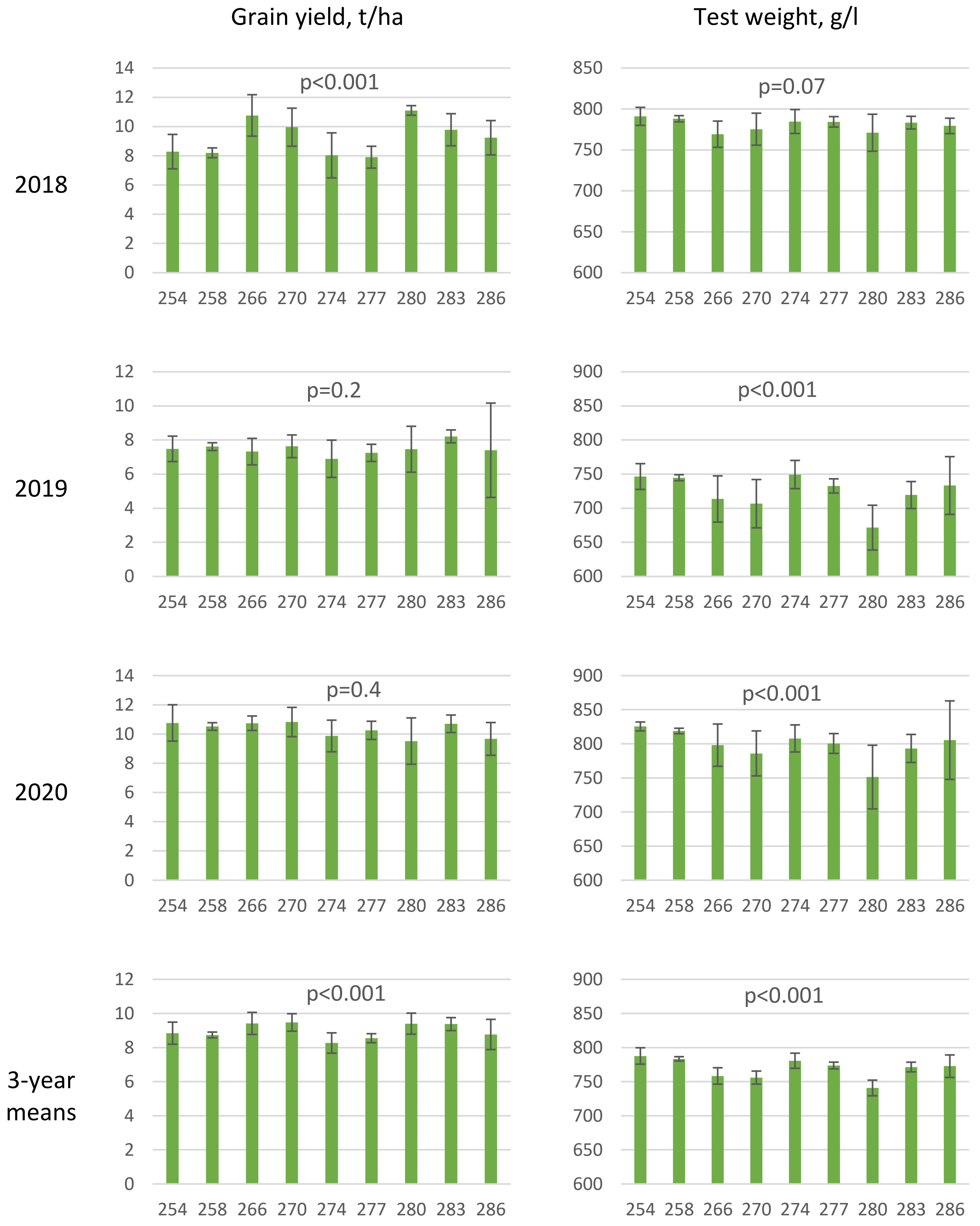

Figure S3. Yield and test weight for wheat accessions differing in size of a microsatellite in the 5'UTR of the *GRF3-2A* gene during three years (2018-2020) at Krasnodar. Sizes of the microsatellite marker GRF3-2AD-SSR detected by fragment analysis are indicated on *x*-axis. Bars indicate 95% confidence intervals. The p-values are calculated by one-way analysis of variance (ANOVA) and Fisher's F-test.

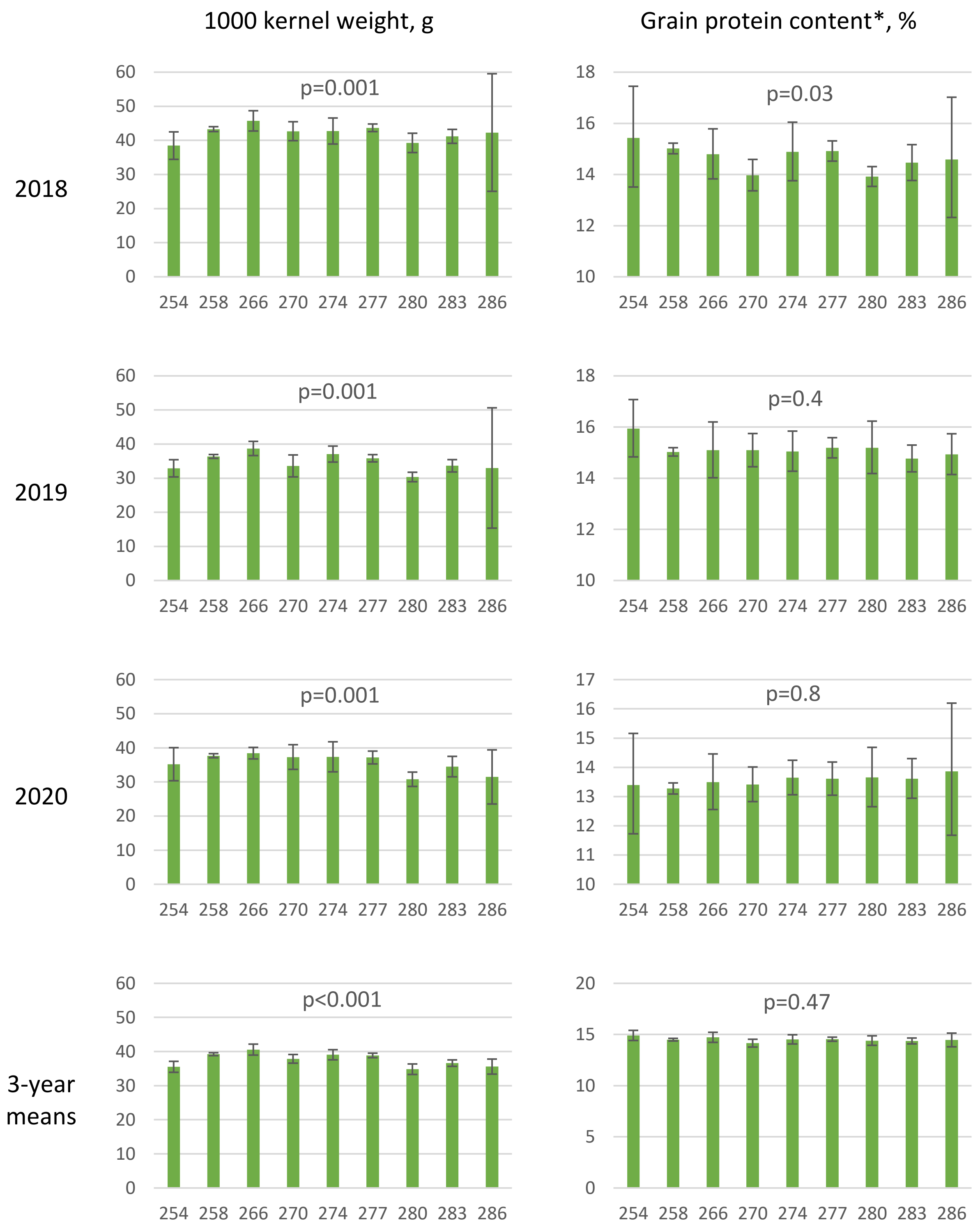

Figure S4. 1000 kernel weight and grain protein content for wheat accessions differing in size of the microsatellite in the 5'UTR of the *GRF3-2A* gene during three years (2018-2020) at Krasnodar. Sizes of the microsatellite marker GRF3-2AD-SSR detected by fragment analysis are indicated on *x*-axis. Bars indicate 95% confidence intervals. The p-values are calculated by one-way analysis of variance (ANOVA) and Fisher's F-test. Values of the grain protein content were transformed using $arcsin(\sqrt{x/100})$ before ANOVA. The reverse-transformed means and boundaries of confidence intervals are shown on histogram.
