# Supplementary material for: Alleles of the GRF3-2A Gene in Wheat and Their Agronomic Value
Source: Int J Mol Sci. 2021 Nov 16;22(22):12376. doi: 10.3390/ijms222212376 (PMC8622619; doi:10.3390/ijms222212376)
Supplement: Supplementary file 1 [file ijms-22-12376-s001.zip › Figures S5-S6 Weather conditions.pdf]

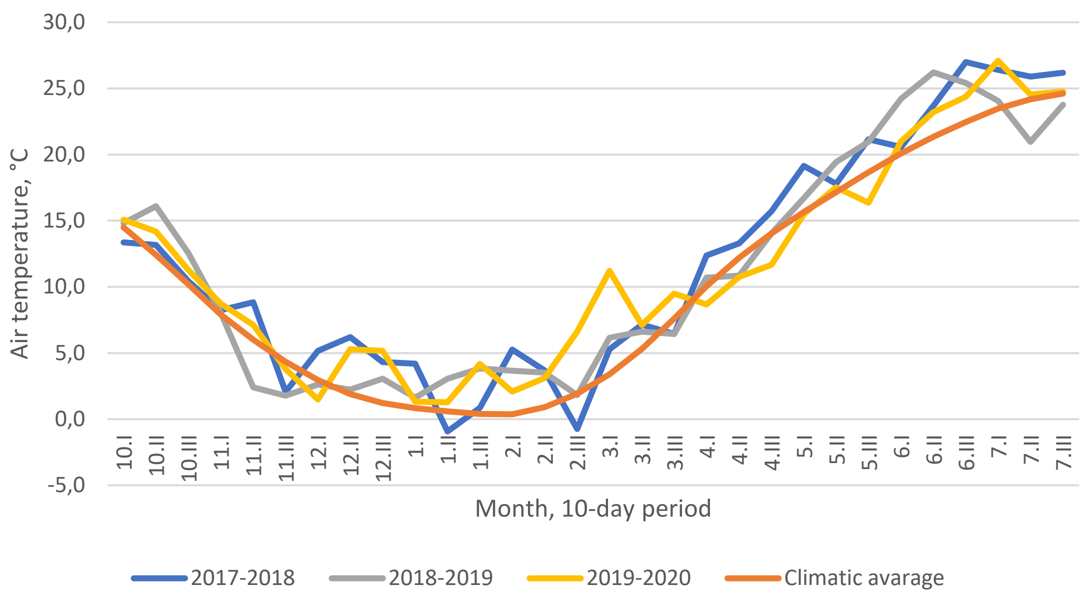

Figure S5. Mean temperature for 10-day periods from sowing (17-21 October) to harvesting of winter wheat at Krasnodar.

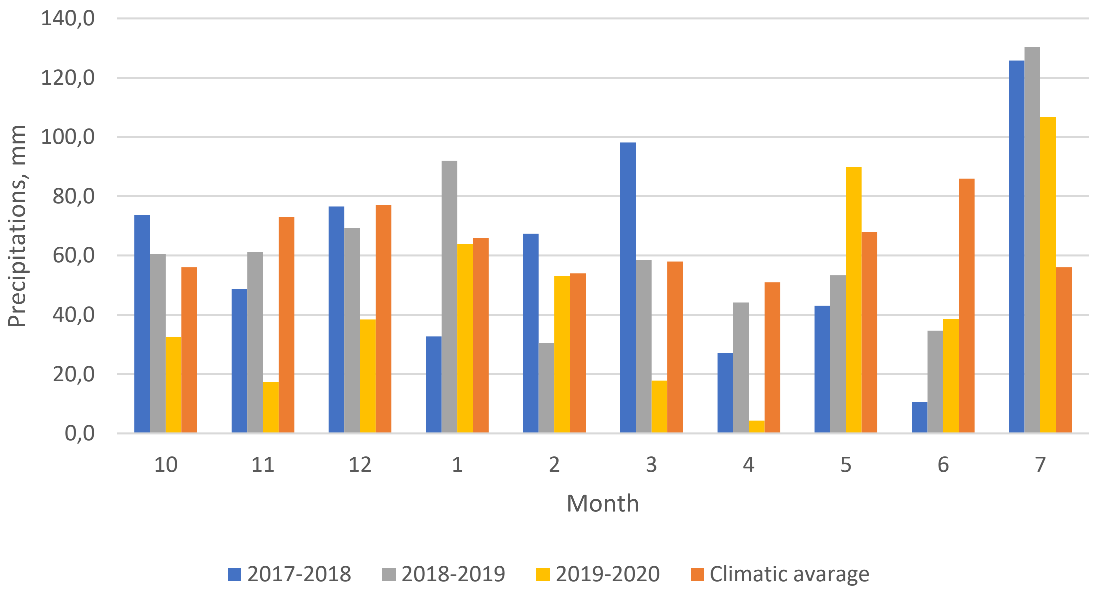

Figure S6. Monthly sums of precipitations from sowing (17-21 October) till harvesting of winter wheat at Krasnodar.
