# Supplementary material for: Alleles of the GRF3-2A Gene in Wheat and Their Agronomic Value
Source: Int J Mol Sci. 2021 Nov 16;22(22):12376. doi: 10.3390/ijms222212376 (PMC8622619; doi:10.3390/ijms222212376)
Supplement: Supplementary file 1 [file ijms-22-12376-s001.zip › Table S1 Accessions for gene sequencing.pdf]

Table S1. Bread wheat (*Triticum aestivum* L.) cultivars and breeding lines in which *TaGRF3-2A* gene was PCR-amplified and sequenced

Accession name	Growth habit	Country of origin	Locality	Reference
Alekseich	winter	Russia	Krasnodar	https://reestr.gossortrf.ru/sorts/8556858/
Al-Murug	spring	Iraq		https://doi.org/10.22363/2312-797X-2019-14-3-196-208
Altigo	winter	France		http://wheatpedigree.net/sort/show/110689
Doka	winter	Russia	Krasnodar	http://wheatpedigree.net/sort/show/81808
Fisht	winter	Russia	Krasnodar	http://wheatpedigree.net/sort/show/76190
Grom	winter	Russia	Krasnodar	http://wheatpedigree.net/sort/show/85804
Iraq	spring	Iraq		https://doi.org/10.22363/2312-797X-2019-14-3-196-208
L.2877k58	winter	Russia	Krasnodar	
Novosibirskaya 67	spring	Russia	Novosibirsk	http://wheatpedigree.net/sort/show/45416
Proton	winter	Russia	Krasnodar	http://wheatpedigree.net/sort/show/88961
Romi	spring	Spain		http://wheatpedigree.net/sort/show/52824
Saratovskaya 29	spring	Russia	Saratov	http://wheatpedigree.net/sort/show/56274
Sila	winter	Russia	Krasnodar	http://wheatpedigree.net/sort/show/85820
Stan	winter	Russia	Krasnodar	http://wheatpedigree.net/sort/show/117035
Starshina	winter	Russia	Krasnodar	http://wheatpedigree.net/sort/show/79122
Tamuz 3	spring	Iraq		http://wheatpedigree.net/sort/show/71100
Vassa	winter	Russia	Krasnodar	http://wheatpedigree.net/sort/show/88955
Velena	intermediate	Russia	Krasnodar	https://reestr.gossortrf.ru/sorts/8654904/
Vid	winter	Russia	Krasnodar	https://reestr.gossortrf.ru/sorts/8556856/
