# Supplementary material for: Alleles of the GRF3-2A Gene in Wheat and Their Agronomic Value
Source: Int J Mol Sci. 2021 Nov 16;22(22):12376. doi: 10.3390/ijms222212376 (PMC8622619; doi:10.3390/ijms222212376)
Supplement: Supplementary file 1 [file ijms-22-12376-s001.zip › Table S4 Accessions for genotyping.pdf]

Table S4. Wheat accessions used for *GRF3-2A* genotyping using molecular markers

No	Accession name	Species[1]	Growth habit	Country of origin	Locality	Reference, pedigree
1	06380AR83	*T. aest.*	winter	Russia	Krasnodar	
2	4023W5	*T. aest.*	winter	Russia	Krasnodar	
3	5577h56G10	*T. aest.*	winter	Russia	Krasnodar	
4	609sv25	*T. aest.*	winter	Russia	Krasnodar	
5	7675h34-23	*T. aest.*	winter	Russia	Krasnodar	
6	7675h34-34	*T. aest.*	winter	Russia	Krasnodar	
7	ACPG 154	*T. aest.*	winter	Russia	Moscow	https://reestr.gossortrf.ru/sorts/8260623/
8	Adel	*T. aest.*	winter	Russia	Krasnodar	http://wheatpedigree.net/sort/show/115868
9	Afina	*T. aest.*	intermediate	Russia	Krasnodar	http://wheatpedigree.net/sort/show/83082
10	Aivina	*T. aest.*	winter	Russia	Krasnodar	http://wheatpedigree.net/sort/show/85764
11	Alekseich	*T. aest.*	winter	Russia	Krasnodar	https://reestr.gossortrf.ru/sorts/8556858/
12	Altigo	*T. aest.*	winter	France		http://wheatpedigree.net/sort/show/110689
13	Anastasiya	*T. aest.*	winter	Russia	Saratov	https://reestr.gossortrf.ru/sorts/8458032/
14	Antonina	*T. aest.*	winter	Russia	Krasnodar	http://wheatpedigree.net/sort/show/117445
15	Apache	*T. aest.*	winter	France		http://wheatpedigree.net/sort/show/79265
16	Armada	*T. aest.*	winter	Russia	Stavropol	https://reestr.gossortrf.ru/sorts/8356459/
17	Arsenal	*T. aest.*	winter	Russia	Stavropol	https://reestr.gossortrf.ru/sorts/8354653/
18	Astarta	*T. aest.*	winter	Ukraine	Kiev	http://wheatpedigree.net/sort/show/117865
19	Bagrat	*T. aest.*	winter	Russia	Krasnodar	http://wheatpedigree.net/sort/show/116998
20	Balitus	*T. aest.*	winter	Austria		https://reestr.gossortrf.ru/sorts/8556863/
21	Barok	*T. aest.*	winter	France		
22	Bazis	*T. aest.*	winter	Russia	Samara	https://reestr.gossortrf.ru/sorts/8558188/
23	Bet B 8 14	*T. aest.*	winter	Bulgaria	Sofia	https://reestr.gossortrf.ru/sorts/8260772/
24	Bezenchukskaya 380	*T. aest.*	winter	Russia	Samara	http://wheatpedigree.net/sort/show/6992
25	Bezostaya 1	*T. aest.*	winter	Russia	Krasnodar	http://wheatpedigree.net/sort/show/7007
26	Bezostaya 100	*T. aest.*	winter	Russia	Krasnodar	https://reestr.gossortrf.ru/sorts/8556857/

27	Bis	*T. aest.*	winter	Russia	Vladimir	http://wheatpedigree.net/sort/show/112371
28	Bodryi	*T. aest.*	winter	Russia	Moscow	https://reestr.gossortrf.ru/sorts/8354640/
29	Bohemia	*T. aest.*	winter	Czech Republic	Sibřina	http://wheatpedigree.net/sort/show/91679
30	Boyarynya	*T. aest.*	winter	Russia	Rostov	http://wheatpedigree.net/sort/show/117380
31	Brigada	*T. aest.*	winter	Russia	Krasnodar	http://wheatpedigree.net/sort/show/96315
32	BT 2375 p 15	*T. aest.*	winter	unknown		
33	BT 2385 p 15	*T. aest.*	winter	unknown		
34	Butterfly	*T. aest.*	winter	Czech Republic	Sibřina	https://reestr.gossortrf.ru/sorts/8152807/
35	Bylina Dona	*T. aest.*	winter	Russia	Rostov	https://reestr.gossortrf.ru/sorts/8356468/
36	Caphorn	*T. aest.*	winter	UK		http://wheatpedigree.net/sort/show/79045
37	Cheget	*T. aest.*	winter	Russia	Krasnodar	http://wheatpedigree.net/sort/show/83196
38	Chornyava	*T. aest.*	winter	Ukraine	Kiev	http://wheatpedigree.net/sort/show/113665
39	Clarion	*T. aest.*	winter	Germany		http://wheatpedigree.net/sort/show/114338
40	Crimson	*T. aest.*	winter	USA	South-Dakota	http://wheatpedigree.net/sort/show/72051
41	Dagmar	*T. aest.*	winter	Czech Republic		http://wheatpedigree.net/sort/show/113244
42	Desamo	*T. aest.*	winter	Germany		http://wheatpedigree.net/sort/show/118984
43	Deya	*T. aest.*	winter	Russia	Krasnodar	http://wheatpedigree.net/sort/show/75797
44	Dmitrii	*T. aest.*	winter	Russia	Krasnodar	http://wheatpedigree.net/sort/show/88957
45	Dolya	*T. aest.*	winter	Russia	Krasnodar	http://wheatpedigree.net/sort/show/115870
46	Don 93	*T. aest.*	winter	Russia	Rostov	http://wheatpedigree.net/sort/show/69604
47	Don 95	*T. aest.*	winter	Russia	Rostov	http://wheatpedigree.net/sort/show/69913
48	Donmira	*T. aest.*	winter	Russia	Rostov	https://reestr.gossortrf.ru/sorts/8354656/
49	Donna	*T. aest.*	winter	Russia	Rostov	http://wheatpedigree.net/sort/show/92274
50	Donskaya Lira	*T. aest.*	winter	Russia	Rostov	http://wheatpedigree.net/sort/show/88958
51	Donskaya Polukarlikovaya	*T. aest.*	winter	Russia	Rostov	http://wheatpedigree.net/sort/show/16895
52	Donskaya Step'	*T. aest.*	winter	Russia	Rostov	https://reestr.gossortrf.ru/sorts/8260596/
53	Donskaya Yubileinaya	*T. aest.*	winter	Russia	Rostov	http://wheatpedigree.net/sort/show/16896
54	Donstar	*T. aest.*	winter	Russia	Rostov	http://wheatpedigree.net/sort/show/114157

55	DSV-11-13 (Aspect)	*T. aest.*	winter	Germany	Lippstadt	http://wheatpedigree.net/sort/show/79510
56	Duplet	*T. aest.*	winter	Russia	Krasnodar	https://reestr.gossortrf.ru/sorts/8559198/
57	Ekspromt	*T. aest.*	winter	Ukraine	Kiev	http://wheatpedigree.net/sort/show/79912
58	Eremeevna	*T. sphaer.*	winter	Russia	Krasnodar	http://wheatpedigree.net/sort/show/117048
59	Ermak	*T. aest.*	winter	Russia	Rostov	http://wheatpedigree.net/sort/show/78205
60	Esaul	*T. aest.*	winter	Russia	Krasnodar	http://wheatpedigree.net/sort/show/82560
61	Etnos	*T. aest.*	winter	Russia	Krasnodar	http://wheatpedigree.net/sort/show/96333
62	Euclide	*T. aest.*	winter	France		http://wheatpedigree.net/sort/show/119016
63	Favoritka	*T. aest.*	winter	Ukraine	Kiev	http://wheatpedigree.net/sort/show/83142
64	Fenomen	*T. aest.*	winter	Poland	Łochów	
65	Flamenko	*T. aest.*	winter	France		
66	Fontanka	*T. aest.*	winter	Ukraine		https://reestr.gossortrf.ru/sorts/8757442/
67	Fortuna	*T. aest.*	winter	Russia	Krasnodar	http://wheatpedigree.net/sort/show/82576
68	Gileya	*T. aest.*	winter	Ukraine	Kiev	http://wheatpedigree.net/sort/show/117879
69	Gordian	*T. aest.*	winter	Germany		http://wheatpedigree.net/sort/show/119044
70	Graf	*T. aest.*	winter	Russia	Krasnodar	https://reestr.gossortrf.ru/sorts/8559195/
71	Gratsiya	*T. aest.*	winter	Russia	Krasnodar	http://wheatpedigree.net/sort/show/83086
72	Grom	*T. aest.*	winter	Russia	Krasnodar	http://wheatpedigree.net/sort/show/85804
73	Gubernator Dona	*T. aest.*	winter	Russia	Rostov	http://wheatpedigree.net/sort/show/88159
74	Gurt	*T. aest.*	winter	Russia	Krasnodar	http://wheatpedigree.net/sort/show/118485
75	Hadm 25612-02	*T. aest.*	winter	Germany	Hadmersleben	
76	Inea	*T. aest.*	winter	Russia	Moscow	https://reestr.gossortrf.ru/sorts/8152818/
77	Iridium	*T. aest.*	winter	France		http://wheatpedigree.net/sort/show/110759
78	Irishka	*T. aest.*	winter	Russia	Krasnodar	http://wheatpedigree.net/sort/show/85768
79	Iststar (Lipetskaya zvezda)	*T. aest.*	winter	Germany		https://reestr.gossortrf.ru/sorts/8556877/
80	Julia	*T. aest.*	winter	Romania		http://wheatpedigree.net/sort/show/27862
81	Kalym	*T. aest.*	winter	Russia	Krasnodar	http://wheatpedigree.net/sort/show/88959
82	Karavan	*T. aest.*	intermediate	Russia	Krasnodar	https://reestr.gossortrf.ru/sorts/8558567/

83	Karillon	*T. aest.*	winter	unknown		
84	Kasatka	*T. aest.*	winter	unknown		
85	Kavkaz	*T. aest.*	winter	Russia	Krasnodar	http://wheatpedigree.net/sort/show/31167
86	Knyaginya Olga	*T. aest.*	winter	Ukraine	Odessa	http://wheatpedigree.net/sort/show/96617
87	Kollega	*T. aest.*	winter	Russia	Krasnodar	http://wheatpedigree.net/sort/show/83127
88	Kometa	*T. aest.*	winter	Poland	Krakow	https://reestr.gossortrf.ru/sorts/8260948/
89	Krasa Dona	*T. aest.*	winter	Russia	Rostov	https://reestr.gossortrf.ru/sorts/8559203/
90	Krasnodarskaya 6	*T. aest.*	winter	Russia	Krasnodar	http://wheatpedigree.net/sort/show/32803
91	Krasnodarskaya 99	*T. aest.*	winter	Russia	Krasnodar	http://wheatpedigree.net/sort/show/77550
92	Krasnoobskaya oz.	*T. aest.*	winter	Russia	Novosibirsk	https://reestr.gossortrf.ru/sorts/8261651/
93	Kurs	*T. aest.*	winter	Russia	Krasnodar	http://wheatpedigree.net/sort/show/58819
94	KWS Barrel	*T. aest.*	winter	UK		http://wheatpedigree.net/sort/show/117959
95	KWS Siskin	*T. aest.*	winter	UK		http://wheatpedigree.net/sort/show/117962
96	KWS Zyatt	*T. aest.*	winter	UK		
97	L.2168k4-3	*T. aest.*	winter	Russia	Krasnodar	
98	L.2293k	*T. aest.*	winter	Russia	Krasnodar	
99	L.2612k2-1	*T. aest.*	winter	Russia	Krasnodar	
100	L.2877k58	*T. aest.*	winter	Russia	Krasnodar	
101	L.3124k4	*T. aest.*	winter	Russia	Krasnodar	
102	L.3244k29-1	*T. aest.*	winter	Russia	Krasnodar	
103	L.3451k	*T. aest.*	winter	Russia	Krasnodar	
104	L.3947k2-4	*T. aest.*	winter	Russia	Krasnodar	
105	L.4648k12	*T. aest.*	winter	Russia	Krasnodar	
106	Laureat	*T. aest.*	winter	Russia	Krasnodar	http://wheatpedigree.net/sort/show/97287
107	Lebed	*T. aest.*	winter	Russia	Krasnodar	http://wheatpedigree.net/sort/show/85769
108	Lgovskaya 4	*T. aest.*	winter	Russia	Kursk	http://wheatpedigree.net/sort/show/82501
109	Liga 1	*T. aest.*	winter	Russia	Krasnodar	http://wheatpedigree.net/sort/show/85770
110	LITERA	*T. aest.*	winter	Romania		http://wheatpedigree.net/sort/show/112008

111	Lut.1234	*T. aest.*	winter	Russia	Krasnodar	
112	Lut.3164h16-23-14	*T. aest.*	winter	Russia	Krasnodar	
113	Lut.4498h138G7	*T. aest.*	winter	Russia	Krasnodar	
114	LYRIK	*T. aest.*	winter	France		
115	Markiz	*T. aest.*	winter	Russia	Krasnodar	
116	Menestrel	*T. aest.*	winter	France		http://wheatpedigree.net/sort/show/110769
117	MIRELA	*T. aest.*	winter	Czech Republic	Bohemia	http://wheatpedigree.net/sort/show/41719
118	Mironovskaya 808	*T. aest.*	winter	Ukraine	Kiev	http://wheatpedigree.net/sort/show/41756
119	Mone	*T. aest.*	winter	France		http://wheatpedigree.net/sort/show/42149
120	Morozko	*T. aest.*	winter	Russia	Krasnodar	http://wheatpedigree.net/sort/show/117030
121	Moskovskaya 40	*T. aest.*	winter	Russia	Moscow	http://wheatpedigree.net/sort/show/88928
122	Moskovskaya 82	*T. aest.*	winter	Russia	Moscow	https://reestr.gossortrf.ru/sorts/8262641/
123	Moskvich	*T. aest.*	winter	Russia	Krasnodar	http://wheatpedigree.net/sort/show/82539
124	Musik	*T. aest.*	winter	France		
125	Mv Nador	*T. aest.*	winter	Hungary		http://wheatpedigree.net/sort/show/116412
126	Mv Nemere	*T. aest.*	winter	Hungary		http://wheatpedigree.net/sort/show/119141
127	Mv Vekni	*T. aest.*	winter	Hungary		http://wheatpedigree.net/sort/show/88148
128	Nakhodka	*T. aest.*	winter	Russia	Rostov	http://wheatpedigree.net/sort/show/117031
129	NEKOTA	*T. aest.*	winter	USA	South-Dakota	http://wheatpedigree.net/sort/show/71003
130	Nemchinovskaya 17	*T. aest.*	winter	Russia	Moscow	http://wheatpedigree.net/sort/show/113660
131	Nemchinovskaya 24	*T. aest.*	winter	Russia	Moscow	http://wheatpedigree.net/sort/show/81229
132	Nemchinovskaya 57	*T. aest.*	winter	Russia	Moscow	http://wheatpedigree.net/sort/show/85744
133	Nemchinovskaya 85	*T. aest.*	winter	Russia	Moscow	https://reestr.gossortrf.ru/sorts/8262640/
134	NIC 11 11637 D	*T. aest.*	winter	France	Clermont-Ferrand	https://reestr.gossortrf.ru/sorts/8559179/
135	Nikifor	*T. aest.*	winter	Romania		http://wheatpedigree.net/sort/show/115661
136	Nota	*T. aest.*	winter	Russia	Krasnodar	http://wheatpedigree.net/sort/show/82572
137	Novosibirskaya 32	*T. aest.*	winter	Russia	Novosibirsk	http://wheatpedigree.net/sort/show/82398

138	NYCKOR	*T. aest.*	winter			
139	Odesskaya 200	*T. aest.*	winter	Ukraine	Odessa	http://wheatpedigree.net/sort/show/82520
140	Olkhon	*T. aest.*	winter	Russia	Krasnodar	http://wheatpedigree.net/sort/show/96206
141	Ordynka	*T. sphaer.*	winter	Russia	Krasnodar	https://reestr.gossortrf.ru/sorts/8260617/
142	Pamyat	*T. aest.*	winter	Russia	Krasnodar	http://wheatpedigree.net/sort/show/78221
143	Paritet	*T. aest.*	winter	Russia	Stavropol	https://reestr.gossortrf.ru/sorts/8356460/
144	Platin	*T. aest.*	winter	Poland		
145	Praskovya	*T. sphaer.*	winter	Russia	Krasnodar	http://wheatpedigree.net/sort/show/113672
146	Proton	*T. aest.*	winter	Russia	Krasnodar	http://wheatpedigree.net/sort/show/88961
147	Proza	*T. aest.*	winter	Russia	Vladimir	http://wheatpedigree.net/sort/show/110921
148	Rossyp	*T. aest.*	winter	Russia	Krasnodar	https://reestr.gossortrf.ru/sorts/8262669/
149	Schit	*T. aest.*	winter	Russia	Stavropol	https://reestr.gossortrf.ru/sorts/8152760/
150	Sila	*T. aest.*	winter	Russia	Krasnodar	http://wheatpedigree.net/sort/show/85820
151	Simonida	*T. aest.*	winter	Yugoslavia	Serbia	http://wheatpedigree.net/sort/show/90607
152	Sineva	*T. aest.*	winter	Russia	Orel	https://reestr.gossortrf.ru/sorts/8354631/
153	Smuglyanka	*T. aest.*	winter	Russia	Saratov	http://wheatpedigree.net/sort/show/69615
154	Snigurka	*T. aest.*	winter	Ukraine	Kiev	http://wheatpedigree.net/sort/show/112229
155	Sodruzhestvo	*T. aest.*	winter	Russia	Barnaul	https://reestr.gossortrf.ru/sorts/8262621/
156	Solovey	*T. aest.*	winter	Russia	Kursk	https://reestr.gossortrf.ru/sorts/8559231/
157	Stan	*T. aest.*	winter	Russia	Krasnodar	http://wheatpedigree.net/sort/show/117035
158	Stanichnaya	*T. aest.*	winter	Russia	Rostov	http://wheatpedigree.net/sort/show/78214
159	Step	*T. aest.*	winter	Russia	Krasnodar	https://reestr.gossortrf.ru/sorts/8559199/
160	Svarog	*T. aest.*	winter	Russia	Krasnodar	https://reestr.gossortrf.ru/sorts/8556853/
161	Tabor	*T. aest.*	winter	Russia	Krasnodar	http://wheatpedigree.net/sort/show/113662
162	Tanais	*T. aest.*	winter	Russia	Rostov	http://wheatpedigree.net/sort/show/82542
163	Tandem	*T. aest.*	winter	USA	South-Dakota	http://wheatpedigree.net/sort/show/69745
164	Tanya	*T. aest.*	winter	Russia	Krasnodar	http://wheatpedigree.net/sort/show/82499
165	Tayfun 7	*T. aest.*	winter	Russia	Stavropol	https://reestr.gossortrf.ru/sorts/8152839/

166	Travitsa	*T. aest.*	winter	Russia	Belgorod	https://reestr.gossortrf.ru/sorts/8152850/
167	Trio	*T. aest.*	winter	Russia	Krasnodar	http://wheatpedigree.net/sort/show/113664
168	Turanus	*T. aest.*	winter	Austria		https://reestr.gossortrf.ru/sorts/8456252/
169	Turnia	*T. aest.*	winter	Poland		http://wheatpedigree.net/sort/show/74657
170	Tvorets	*T. aest.*	winter	Russia	Krasnodar	http://wheatpedigree.net/sort/show/88964
171	Ultra 11	*T. aest.*	winter	Russia	Krasnodar	
172	Ulyasha	*T. aest.*	winter	Russia	Stavropol	https://reestr.gossortrf.ru/sorts/8354709/
173	Urup	*T. aest.*	winter	Russia	Krasnodar	http://wheatpedigree.net/sort/show/116997
174	Utrish	*T. aest.*	winter	Russia	Krasnodar	http://wheatpedigree.net/sort/show/88965
175	Vassa	*T. aest.*	winter	Russia	Krasnodar	http://wheatpedigree.net/sort/show/88955
176	Vekha	*T. aest.*	winter	Russia	Krasnodar	https://reestr.gossortrf.ru/sorts/8556855/
177	Velena	*T. aest.*	intermediate	Russia	Krasnodar	https://reestr.gossortrf.ru/sorts/8654904/
178	Vershina	*T. aest.*	winter	Russia	Krasnodar	http://wheatpedigree.net/sort/show/88956
179	Vid	*T. aest.*	winter	Russia	Krasnodar	https://reestr.gossortrf.ru/sorts/8556856/
180	Videya	*T. aest.*	winter	Russia	Krasnodar	https://reestr.gossortrf.ru/sorts/8458123/
181	Vita	*T. aest.*	winter	Russia	Krasnodar	http://wheatpedigree.net/sort/show/79046
182	Viuga	*T. aest.*	winter	Russia	Samara	https://reestr.gossortrf.ru/sorts/8356347/
183	Viza	*T. aest.*	winter	Russia	Krasnodar	http://wheatpedigree.net/sort/show/82559
184	Volnyi Don	*T. aest.*	winter	Russia	Rostov	https://reestr.gossortrf.ru/sorts/8260593/
185	Volzhskaya Elena	*T. aest.*	winter	Russia	Ulyanovsk	
186	Volzhskaya K	*T. aest.*	winter	Russia	Ulyanovsk	http://wheatpedigree.net/sort/show/82405
187	Xiao Yan 107	*T. aest.*	winter	China		http://wheatpedigree.net/sort/show/68164
188	Yubileinaya 100	*T. aest.*	winter	Russia	Krasnodar	http://wheatpedigree.net/sort/show/80056
189	Yubiley Dona	*T. aest.*	winter	Russia	Rostov	https://reestr.gossortrf.ru/sorts/8262620/
190	Yuka	*T. aest.*	winter	Russia	Krasnodar	http://wheatpedigree.net/sort/show/96322
191	Yumpa	*T. aest.*	winter	Russia	Krasnodar	http://wheatpedigree.net/sort/show/85824
192	Yunona	*T. aest.*	winter	Russia	Krasnodar	http://wheatpedigree.net/sort/show/83090
193	Zhavoronok	*T. aest.*	winter	Russia	Rostov	https://reestr.gossortrf.ru/sorts/8260592/

194	Zhiva	*T. aest.*	winter	Russia	Krasnodar	https://reestr.gossortrf.ru/sorts/8556854/
195	Zhong Pin 1629	*T. aest.*	winter	China		
196	Zimnitsa	*T. aest.*	winter	Russia	Krasnodar	http://wheatpedigree.net/sort/show/85767
197	Zimtra	*T. aest.*	winter	Russia	Krasnodar	http://wheatpedigree.net/sort/show/83126
198	Zina	*T. aest.*	winter	Russia	Stavropol	https://reestr.gossortrf.ru/sorts/8655024/
199	Zolotokolosa	*T. aest.*	winter	Ukraine	Kiev	http://wheatpedigree.net/sort/show/88121

[1] – *T. aest.* = *Triticum aestivum* L. subsp. *aestivum*; *T. sphaer*. = *Triticum aestivum* L. subsp. *sphaerococcum* (Percival) Mac Key
