# Supplementary material for: Alleles of the GRF3-2A Gene in Wheat and Their Agronomic Value
Source: Int J Mol Sci. 2021 Nov 16;22(22):12376. doi: 10.3390/ijms222212376 (PMC8622619; doi:10.3390/ijms222212376)
Supplement: Supplementary file 1 [file ijms-22-12376-s001.zip › Table S6 Accessions with assembled genome.pdf]

Table S6. Wheat accessions, for which a complete genome sequence is available, and which were used for *GRF3-2A* allele discovery

Accession name	Species	Growth habit	Status	Country of origin	Reference (DOI)
ArinaLrFor	*T. aestivum* L. subsp. *aestivum*	winter	breeding material	Switzerland	10.1038/s41586-020-2961-x
Cadenza	*T. aestivum* L. subsp. *aestivum*	spring	cultivar	UK England	10.1038/s41586-020-2961-x
CDC Landmark	*T. aestivum* L. subsp. *aestivum*	spring	cultivar	Canada	10.1038/s41586-020-2961-x
CDC Stanley	*T. aestivum* L. subsp. *aestivum*	spring	cultivar	Canada	10.1038/s41586-020-2961-x
Chinese Spring	*T. aestivum* L. subsp. *aestivum*	spring	landrace	China	10.1126/science.aar7191
Claire	*T. aestivum* L. subsp. *aestivum*	winter	cultivar	UK England	10.1038/s41586-020-2961-x
Jagger	*T. aestivum* L. subsp. *aestivum*	winter	cultivar	USA	10.1038/s41586-020-2961-x
Julius	*T. aestivum* L. subsp. *aestivum*	winter	cultivar	Germany	10.1038/s41586-020-2961-x
Kronos	*T. turgidum* L. subsp. *durum* (Desf.) van Slageren	spring	cultivar	USA	10.3389/fpls.2019.01244
Lancer	*T. aestivum* L. subsp. *aestivum*	spring	cultivar	Australia	10.1038/s41586-020-2961-x
Mace	*T. aestivum* L. subsp. *aestivum*	winter	cultivar	Australia	10.1038/s41586-020-2961-x
Norin 61	*T. aestivum* L. subsp. *aestivum*	winter	cultivar	Japan	10.1038/s41586-020-2961-x
Paragon	*T. aestivum* L. subsp. *aestivum*	spring	cultivar	UK England	10.1038/s41586-020-2961-x
Robigus	*T. aestivum* L. subsp. *aestivum*	winter	cultivar	UK England	10.1038/s41586-020-2961-x
Spelt (PI 190962)	*T. aestivum* L. subsp. *spelta* (L.) Thell.	spring	uncertain	Italy	10.1038/s41586-020-2961-x
SY Mattis	*T. aestivum* L. subsp. *aestivum*	winter	cultivar	France	10.1038/s41586-020-2961-x
Urartu G1812 (PI428198)	*T. urartu* Tumanian ex Gandilyan	winter	wild	Turkey	10.1038/s41586-018-0108-0
Zavitan	*T. turgidum* ssp. *dicoccoides* (Körn.) Thell.	winter	wild	Israel	10.1126/science.aan0032
