# Supplementary material for: Alleles of the GRF3-2A Gene in Wheat and Their Agronomic Value
Source: Int J Mol Sci. 2021 Nov 16;22(22):12376. doi: 10.3390/ijms222212376 (PMC8622619; doi:10.3390/ijms222212376)
Supplement: Supplementary file 1 [file ijms-22-12376-s001.zip › Tables S2-S3 TaGRF3-2A alleles.pdf]

Table S2. *TaGRF3-2A* alleles and GRF3 protein isoforms in wheat accessions

Species	Accession	Allele	Protein isoform	Sequence source*
T. aestivum	Al-Murug	a.2	A	S
T. aestivum	Altigo	a.4	A	S
T. aestivum	ArinaLrFor	a.3	A	G
T. aestivum	Cadenza	a.3	A	G
T. aestivum	CDC Landmark	a.10	A	G
T. aestivum	CDC Stanley	a.14	A	G
T. aestivum	Chinese Spring	a.1	A	G
T. aestivum	Claire	a.11	A	G
T. aestivum	Grom	a.4	A	S
T. aestivum	Iraq	a.7	A	S
T. aestivum	Jagger	a.5	A	G
T. aestivum	Julius	a.3	A	G
T. aestivum	Lancer	a.9	A	G
T. aestivum	Mace	a.5	A	G
T. aestivum	Norin 61	a.15	A	G
T. aestivum	Paragon	a.13	A	G
T. aestivum	Robigus	a.1	A	G
T. aestivum	Romy	a.1	A	S
T. aestivum	Saratovskaya 29	a.8	A	S
T. aestivum	Sila	a.2	A	S
T. aestivum	SY Mattis	a.12	A	G
T. aestivum	Tamuz 3	a.2	A	S
T. aestivum	Velena	a.6	A	S
T. aestivum	Alekseich	b.1	B	S
T. aestivum	Doka	b.1	B	S
T. aestivum	Fisht	b.2	B	S
T. aestivum	L.2877k58	b.4	B	S
T. aestivum	Proton	b.1	B	S
T. aestivum	Stan	b.2	B	S
T. aestivum	Starshina	b.5/b.2	B	S
T. aestivum	Vassa	b.3	B	S
T. aestivum	Vid	b.1	B	S
T. aestivum	Novosibirskaya 67	d.1	D	S
T. dicoccoides	Zavitan (wild emmer wheat)	a.16	A	G
T. durum	Kronos (durum wheat)	a.1	A	G
T. spelta	PI190962 (spelt wheat)	a.17	A	G
T. urartu	PI428198 (einkorn wheat)	c.1	C	G

T. aestivum = *Triticum aestivum* L. subsp. aestivum; *T. sphaerococum* = *Triticum aestivum* L. subsp. *sphaerococcum* (Percival) Mac Key; *T. urartu* = *Triticum urartu* Tumanian ex Gandilyan; *T. dicoccoides* = *Triticum turgidum* ssp. *dicoccoides*; *T. durum* = *Triticum turgidum* L. subsp. *durum* (Desf.) van Slageren; *T. spelta* = *Triticum aestivum* L. subsp. *spelta* (L.) Thell.

* S – the original sequences obtained by the authors (see Materials and methods); G – the sequences found by BLAST search in previously sequenced and assembled genomes.

Table S3. Frequencies of *GRF3-2A* alleles among sequenced[1] wheat accessions

Alleles of *GRF3-2A*	Allele frequencies for			
	all accessions	all bread wheat *(T. aestivum ssp. aestivum)*	bread wheat from Lukyanenko National Grain Center (Krasnodar)	bread wheat from other sources (not from Krasnodar)
a.1	0.11	0.09	0.00	0.14
a.2	0.08	0.09	0.09	0.10
a.3	0.08	0.09	0.00	0.14
a.4	0.05	0.06	0.09	0.05
a.5	0.05	0.06	0.00	0.10
a.6	0.03	0.03	0.09	0.00
a.7	0.03	0.03	0.00	0.05
a.8	0.03	0.03	0.00	0.05
a.9	0.03	0.03	0.00	0.05
a.10	0.03	0.03	0.00	0.05
a.11	0.03	0.03	0.00	0.05
a.12	0.03	0.03	0.00	0.05
a.13	0.03	0.03	0.00	0.05
a.14	0.03	0.03	0.00	0.05
a.15	0.03	0.03	0.00	0.05
a.16	0.03	0.00	0.00	0.00
a.17	0.03	0.00	0.00	0.00
b.1	0.11	0.12	0.36	0.00
b.2	0.07	0.08	0.18	0.00
b.3	0.03	0.03	0.09	0.00
b.4	0.03	0.03	0.09	0.00
b.5	0.01	0.02	0.00	0.00
c.1	0.03	0.00	0.00	0.00
d.1	0.03	0.03	0.00	0.05
Number of accessions	37	33	12	21

[1] – *GRF3-2A* gene was target-sequenced for these accessions or sequence of it was obtained from available genome sequence.
