# Supplementary material for: Alleles of the GRF3-2A Gene in Wheat and Their Agronomic Value
Source: Int J Mol Sci. 2021 Nov 16;22(22):12376. doi: 10.3390/ijms222212376 (PMC8622619; doi:10.3390/ijms222212376)
Supplement: Supplementary file 1 [file ijms-22-12376-s001.zip › Tables S7-S8 Agronomic traits means.pdf]

Table S7. Agronomic traits of winter wheat accessions in connection with c.495G>T polymorphism in *TaGRF3-2A* gene. Means and ±95% confidence intervals are given.

c.495G>T	Year			Mean
	2018	2019	2020	
		**Grain yield, t/ha**		
**G**	8,8±0,4	7,5±0,2	10,2±0,3	8,9±0,2
**T**	8,2±0,4	7,5±0,3	10,3±0,3	8,7±0,2
		**Heading date, days after sowing**		
**G**	198±0,8	203±0,7	205±0,9	202±0,4
**T**	197±0,7	202±0,8	203±0,9	201±0,5
		**Test weight, g/l**		
**G**	783±4	731±7	801±6	771±3
**T**	789±4	745±5	817±4	784±4
		**1000 kernel weight, g**		
**G**	42,4±0,7	34,8±0,6	36,3±0,9	37,8±0,4
**T**	43,3±0,8	36,6±0,7	37,4±0,7	39,1±0,5
		**Grain protein content, %**		
**G**	14,7±0,2	15,1±0,2	13,6±0,2	14,5±0,1
**T**	15,1±0,2	15,1±0,2	13,5±0,2	14,6±0,1
		**Protein yield, t/ha**		
**G**	1,29±0,05	1,14±0,03	1,38±0,03	1,27±0,02
**T**	1,23±0,06	1,14±0,03	1,38±0,03	1,25±0,02

Table S8. Agronomic traits of winter wheat accessions in connection with a microsatellite marker in the 5'UTR of the *TaGRF3-2A* gene. Means and ±95% confidence intervals are given.

GRF-2A-SSR	Year			Mean
	2018	2019	2020	
		**Grain yield, t/ha**		
**254**	8,3±1,2	7,5±0,7	10,8±1,2	8,8±0,6
**258**	8,2±0,3	7,6±0,2	10,5±0,3	8,7±0,2
**266**	10,8±1,4	7,3±0,8	10,7±0,5	9,4±0,6
**270**	10±1,3	7,6±0,7	10,8±1	9,5±0,5
**274**	8±1,5	6,9±1,1	9,9±1,1	8,3±0,6
**277**	7,9±0,7	7,2±0,5	10,3±0,6	8,5±0,3
**280**	11,1±0,3	7,5±1,3	9,5±1,6	9,4±0,6
**283**	9,8±1,1	8,2±0,4	10,7±0,6	9,4±0,4
**286**	9,2±1,2	7,4±2,8	9,7±1,1	8,8±0,9
		**Test weight, g/l**		
**254**	791±11	747±19	825±7	825±7
**258**	788±4	745±4	819±4	819±4
**266**	769±16	714±34	798±31	798±31
**270**	775±20	707±35	786±33	786±33
**274**	785±15	749±21	808±20	808±20
**277**	784±6	733±10	800±15	800±15
**280**	771±23	672±33	751±47	751±47
**283**	783±8	719±20	793±21	793±21
**286**	779±9	733±42	805±58	805±58
		**1000 kernel weight, g**		
**254**	38,5±4	32,9±2,5	35,2±4,8	35,5±1,6
**258**	43,3±0,7	36,4±0,6	37,7±0,6	39,2±0,4
**266**	45,7±3	38,7±2,1	38,4±1,7	40,6±1,6
**270**	42,7±2,8	33,6±3,2	37,3±3,6	37,9±1,3
**274**	42,7±3,8	37,1±2,3	37,4±4,4	39,1±1,5
**277**	43,7±1,1	35,8±1,1	37,2±1,9	38,9±0,7
**280**	39,3±2,8	30,4±1,4	30,8±2,1	34,8±1,5
**283**	41,2±2	33,6±1,8	34,5±3	36,6±0,9
**286**	42,3±17,2	33±17,6	31,5±7,9	35,6±2,2
		**Grain protein content, %**		
**254**	15,4±2	15,9±1,1	13,4±1,8	14,9±0,5
**258**	15±0,2	15±0,2	13,3±0,2	14,5±0,1
**266**	14,8±1	15,1±1,1	13,5±1	14,7±0,5
**270**	14±0,6	15,1±0,7	13,4±0,6	14,2±0,4
**274**	14,9±1,2	15±0,8	13,6±0,6	14,5±0,5
**277**	14,9±0,4	15,2±0,4	13,6±0,6	14,5±0,2
**280**	13,9±0,4	15,2±1	13,7±1	14,4±0,5
**283**	14,5±0,7	14,8±0,5	13,6±0,7	14,4±0,3
**286**	14,6±2,4	14,9±0,8	13,9±2,3	14,5±0,7
